# Current Regulatory Requirements for Biosimilars in Six Member Countries of BRICS-TM: Challenges and Opportunities

**DOI:** 10.3389/fmed.2021.726660

**Published:** 2021-09-09

**Authors:** Hasumati Rahalkar, Alan Sheppard, Gustavo Mendes Lima Santos, Chitralekha Dasgupta, Sonia Mayra Perez-Tapia, Carlos A. Lopez-Morales, Sam Salek

**Affiliations:** ^1^Metina PharmConsulting Pvt. Ltd., Navi Mumbai, India; ^2^School of Life and Medical Sciences, University of Hertfordshire, Hatfield, United Kingdom; ^3^Ascher Resources Ltd., London, United Kingdom; ^4^Brazilian Health Regulatory Agency, Brasilia, Brazil; ^5^Independent Regulatory Consultant, Moscow, Russia; ^6^Unidad de Desarrollo e Investigación en Bioprocesos, Escuela Nacional de Ciencias Biológicas, Instituto Politécnico Nacional, Mexico City, Mexico; ^7^Institute of Medicines Development, Cardiff, United Kingdom

**Keywords:** BRICS-TM, biosimilar, emerging economies, marketing authorisation, regulatory agency

## Abstract

**Background:** The aim of the study was to identify, interpret, and compare the current perspectives of regulatory agencies in six member countries of BRICS-TM (Brazil, Russia, India, China, South Africa, Turkey, and Mexico) on the different criteria used for biosimilar development and marketing authorisation process.

**Methods:** A semi-quantitative questionnaire was developed covering the organisation of agency, biosimilar development criteria and marketing authorisation process and sent to seven regulatory agencies covering the BRICS-TM countries. All data was kept anonymous and confidential. Data processing and analysis was carried out; descriptive statistics were used for quantitative data and content analysis was employed to generate themes for qualitative data.

**Results:** Out of the seven regulatory agencies included in the study, six representatives provided the responses. The perspectives of these six regulatory agencies varied on a number of aspects relating to the review criteria for biosimilar development and licencing process. The most prevalent model for data assessment is the “full review” of a marketing authorisation application. There is lack of a standard approach across the agencies on sourcing of the reference biological product, *in vivo* toxicity studies and confirmatory clinical studies. Most agencies restrict interaction with biosimilar developers and any scientific advice is non-binding. The marketing authorisation approval depends on scientific assessment of the dossier, sample analysis and GMP certification. The agencies do not issue any public assessment report specifying the summary basis of biosimilar approval.

**Conclusion:** Regulatory agencies across the six emerging economies are steadily improving the regulatory mechanism in the area of biosimilars. However, there remains scope for increasing the effectiveness and efficiency of the processes by encouraging open and transparent interaction with developers, adopting a flexible approach toward accepting advanced analytical data in lieu of clinical studies and enhancing regulatory reliance amongst agencies. This will help to simplify the new biosimilar development programmes and make them more cost-effective.

## Introduction

Emerging economies represent 70% of the world population accounting for a 31% share of global GDP and more than 30% of pharmaceutical spending (1). In addition, they account for one-third of the global growth in drug demand, with annual growth rate of 5–8% (2). Biosimilars, which account for 28% of the global pharmaceutical market have the potential to significantly boost treatment options and hence are expected to play an important role in the pharmaceutical market (3). Emerging economies with low biologic-treatment rates and affordability barriers present attractive opportunities for biosimilars (4). However, marketing authorisations of these much-needed products are often delayed as manufacturers face challenges of multiple regulatory requirements to register products in different countries (5, 6). It is encouraging to note that the regulatory approval pathways for biosimilars applied by the major regulatory agencies worldwide are, to a broad degree, scientifically aligned (7). However, owing to regional differences in healthcare priorities, policies, and resources, some important regulatory inconsistencies are evident in emerging economies. Some of these challenges such as lack of step wise approach, difference in selection and sourcing of Reference Biological Product (RBP), regulatory expectations of clinical efficacy trial design and lack of transparency toward interchangeability, switching and substitution norms, have been identified (8). Inevitably, lack of standardised regulatory processes would hamper the growth of biosimilars in these countries (9). Thus, it is of paramount importance to evaluate the framework for biosimilar development and approval processes in these emerging economies.

In the last 2 decades, the mature regulatory agencies, in particular in ICH jurisdictions, have made significant progress toward establishing, revising, and updating biosimilar guidelines to match the dynamic innovation in biotechnology. However, there remains scope for improvement in establishing regional standardisation for regulatory requirements of biosimilar development and approval process.

The main objectives of the study were to:

- evaluate and compare technical capabilities of the six regulatory agencies of the emerging economies in the area of biosimilars,
- identify similarities and differences in regulatory requirements of biosimilar development criteria i.e., biosimilarity principles, comparative studies including physicochemical characterisation, non-clinical, and clinical studies,
- evaluate and compare “must submit documents” as part of biosimilar application for marketing authorisation in these six emerging economies,
- map the biosimilar marketing authorisation approval pathway specifically for key milestones, scientific advice meetings, clinical trial mandates, and backlogs.

## Methods

### Study Participants

The regulatory authorities included in this study were those which are part of the BRICS-TM grouping. This refers to the countries of Brazil, Russia, India, China, South Africa, Turkey, and Mexico deemed to be developing countries at a similar stage of newly advanced economic development, on their way to becoming developed countries and also known for their significant influence on regional affairs. It was initially developed as BRICS and since 2009, their governments have met annually at formal summits. Russia hosted the most recent, 12th BRICS-TM summit on 17 November 2020, virtually due to the COVID-19 pandemic (10–12). Therefore, the regulatory authorities of all seven countries were invited to take part in the study.

The potential study participants were identified via each respective authority's general email addresses obtained from agency websites, LinkedIn, the research team's personal contacts, ex-employee, and local leading regulatory consultants for each authority. They were selected based on their work experience in the biologic or biosimilar division of the authority, having held a position as a general manager or above or a leading regulatory consultant with a close working relationship with the relevant authority in the biosimilar space. They were sent an electronic mail with brief information about project and the questionnaire, the objective of the study, the number of authorities to be included and requesting their agreement to participate in the study.

Responses and conditions of acceptance were different across all seven authorities. It took ~18 months to receive agreement from two agencies i.e., Agência Nacional de Vigilância Sanitária (ANVISA), the Brazilian Health Regulatory Agency, Brazil, and the South African Health Products Regulatory Agency (SAHPRA). The respondents from the Central Drug Standards Control Organisation (CDSCO), India agreed to participate on anonymity and the Türkiye Ilaç ve Tibbi Cihaz Kurumu (TITCK), Turkish Medicines and Medical Device Agency data was gathered from public sources of the Agency such as Activity report, official website, and Agency's publicity manual. Two agencies including the Russian MoH and Comisión Federal para la Protección contra Riesgos Sanitarios (COFEPRIS), the Federal Commission for the Protection Against Sanitary Risks, Mexico did not respond to the letter of invitation. Consequently, local senior regulatory consultants were engaged as proxy for Russia and Mexico. Despite tremendous efforts to establish direct contact with the National Medical Products Administration (NMPA) of China or via local Chinese regulatory consultants, the outcome was unsuccessful.

On receipt of agreement to participate from the recruited six countries, the self-administered Biosimilar Development, Evaluation, and Authorisation (BDEA) questionnaire was sent via email for completion by the respective authorities. This was followed up by a face-to-face or virtual meetings after receipt of the completed questionnaire. Such meetings were arranged to understand and further interpret the respective agency's view and to verify the validity of the responses to the questionnaire. In addition, copies of the relevant guidelines were requested as part of the questionnaire to verify the responses and to correlate the actual regulatory requirements. This phase of data collection period took place between March and October 2020.

### Measurement Tool

A semi-quantitative questionnaire, Biosimilar Development, Evaluation, and Authorisation (BDEA) was developed (in English) (Supplementary Material). This was based on slight modification of the Centre for Innovation in Regulatory Science (CIRS) questionnaire (13) and information from secondary research in order to map the regulatory processes existing within agencies (8). In addition, expert inputs were received, and the initial drafts were prepared based on inputs from Biologic and Radiopharmaceutical Drugs Directorate (BRDD) – Health Canada, Turkish Medicines, and Medical Device Agency (TITCK) and CIRS. Since the questionnaire was initially developed for small molecules, the modifications were introduced to make it biosimilar-specific. The BDEA was further improved based on pilot validation performed by the Regulatory Authority of Medicines, Equipment and Medical Device (CECMED), Cuba.

### Data Collection

Data for the comparator authorities was collected in 2019–2020. The BDEA questionnaire which standardises the review process allowing key milestones, activities and practises of the seven regulatory authorities to be identified was completed by a senior member of the biosimilar licencing division and validated by the head of the division/authority. The final version of the BDEA questionnaire dated March 2020, consists of 35 pages and the questions are grouped under 22 categories and grouped into three major sections as follows:

*Part I - Organisation of the agency -* This part of the BDEA questionnaire consist of current agency structure, resources in the biosimilar domain, and types of review models i.e., review models employed for scientific assessment (Table 1), level of data required, and extent of assessment of the data as well as reliance on other authorities, if applicable.
*Part II – Agency's view on biosimilar development criteria -* This part covers questions pertaining to biosimilarity principle, selection of RBP, comprehensive comparability criteria including physico-chemical, non-clinical, and clinical studies and “must submit” documents as part of a biosimilar marketing authorisation application.
*Part III – Marketing authorisation approval pathway -* This part presents questions with regards to key milestones i.e., the process of assessment starting from receipt of the dossier, validation/screening, the number of cycles of scientific assessments including the questions to the sponsor/applicant, expert registration committee meetings to the final decision on approval or refusal of a biosimilar for registration. A standardised process map, developed based on the experience of studying established and regulatory agencies of the emerging economies, was embedded in the questionnaire.

**Table 1:** Models of regulatory review.

**Type**	**Title**	**Definition**
I	Verification of Marketing Authorisation Approval Application	• Importing agency “verifies” that the product intended for local sale has been duly registered as declared in the application. • Used to reduce duplication of efforts by agreeing that the importing country will allow certain products to be marketed locally once they have been authorised by one or more reference agencies, elsewhere. • Product characteristics and prescribing information for local marketing conforms to that agreed in the reference authorisation.
II	Abridged review of Marketing Authorisation Approval Application	• Conserves resources by not re-assessing all scientific supporting data that has been reviewed and accepted by reference agency but includes an “abridged” independent review of the product in terms of its use taking into consideration local cultural and environmental factors. • Includes a review of the biopharmaceutical (CMC) data in relation to climatic conditions and a benefit-risk assessment in relation to use in the local ethnic population, medical practise/culture, and patterns of disease. • Approval by a reference agency is a prerequisite before the local authorisation can be granted.
III	Full review of Marketing Authorisation Approval Application	• Suitable resources available including access to appropriate internal and external experts, to carry out a “full” review and evaluation of the supporting scientific data (quality, non-clinical, clinical) for a major application.

*CMC, Chemistry, Manufacturing, and Control*.

### Data Processing and Analysis

Data processing and analysis was carried out using Microsoft excel; descriptive statistics was used for quantitative data and content analysis was employed to generate themes and sub-themes for qualitative data.

### Ethics Approval

The study has been approved by the Health, Science, Engineering and Technology ECDA, University of Hertfordshire [Reference Protocol number: aLMS/PGR/UH/03332(1)].

## Results

For the purpose of clarity, the results will be presented in three parts:

Part I – Organisation of agency;
Part II – Biosimilar development criteria; and
Part III – Marketing authorisation process.

### Demographic Characteristics of the Study Participants

Out of the seven regulatory agencies invited to take part in the study, four agencies, ANVISA (Brazil), SAHPRA (South Africa), CDSCO (India), and TITCK (Turkey) agreed to take part and completed the questionnaire. Leading regulatory consultants working closely with the agencies for biosimilar medicines from Russia and Mexico also participated in the study. However, multiple efforts to reach NMPA (China) either directly or via regulatory experts were unsuccessful. The individuals who completed the questionnaire held senior positions (general manager or above) within the biologic divisions of their regulatory authority. The regulatory consultants were Chief Executive Officers (CEO) of their respective consulting firms.

### Part I - Organisation of Agency

This provided information on agency size and the strength of the biological division including the number of internal assessors with their minimum qualifications and details on support obtained from external assessors or committees (Table 2).

**Table 2:** Organisation of the six regulatory agencies of the BRICS-TM.

	**ANVISA (Brazil)**	**RUSSIA MoH**	**CDSCO (India)**	**SAHPRA (South Africa)**	**TITCK (Turkey)**	**COFEPRIS (Mexico)**
Total agency staff	1,500	930	1,500	>200	1,172	2,000
**Resource allocation in Biologic/Biosimilar division**						
Total staff	24	Not defined^*^	30	10	No information available	20
Number of reviewers	24	Not defined^*^	8	5	No information available	13
Capacity (%)	1.6	Not applicable	2	5	Not applicable	1
**Internal assessors**						
Qualification	Ph.D.	M.Sc. to Ph.D.	M. Pharm^#^	4-year degree to Masters	Experienced, M.Sc., PhD	Bachelor's degree
Segregation by expertise	CMC, Non-clinical, Clinical, Other scientists	No information available	CMC, Non-clinical, Clinical, Microbiologist, Statistician, Assistant Drug Controllers	CMC, Clinical, Microbiologist	CMC, Non-clinical, Clinical, Microbiologist Other scientists, Project manager	CMC, Non-clinical, Clinical, Project manager
**External assessors**						
Support received	No	No information available	Yes	Yes	Yes	Yes
Area of expertise	Not applicable	Not applicable	Non-clinical, Clinical	CMC, Non-clinical, Clinical	CMC, Non-clinical, Clinical	CMC, Non-clinical, Clinical
Biosimilar advisory committee	No	No information available	SEC, RCGM DBT	Biological Medicine Expert Advisory Committee	No	SEPB, NMC
**Data assessment**						
Data Assessment type	Type III	Type III	Type II, III	Type III	Type III	Type I, III
Recognised reference agencies	Not specified	Not specified	EMA, USFDA, BRDD, MHRA, TGA	Not specified	Not specified	EMA, USFDA, TGA

* *No separate biologic division*;

# *RCGM and SEC committee details excluded; CMC, Chemistry, Manufacturing and Control; SEC, Subject Expert Committee; RCGM, Review Committee on Genetic Manipulation; DBT, Department of Biotechnology; SEPB, Subcommittee on evaluation of Biotech Products; NMC, New Molecule Committee*.

**ANVISA -** The capacity of the biological department is around 1.6% in comparison with the total size of the agency. The agency does not engage with external assessors, and the applications are reviewed by qualified internal assessors all of whom hold a PhD as their qualification. The agency relies on type III data assessment (full review of the marketing authorisation application) for most of the applications.

**Russian MoH -** There is no distinction between internal assessors for the review of biological or non-biological marketing authorisation applications, resulting in the same assessors reviewing both types of applications. Product approval is based solely on self-assessment by internal assessors applying type III review model.

**CDSCO -** The capacity of the biological division within CDSCO is 2% representing common internal assessors for the review of all new biological and biosimilar applications. The agency mandates a master's degree in pharmacy as the minimum qualification for internal assessors and takes expert advice from external assessors for the review of both non-clinical and clinical parts of the dossier. The agency has several bodies with different responsibility including: Subject Expert Committee (SEC) for clinical review which comprises external physicians and regulators; the Review Committee for Genetic Manipulation (RCGM) for non-clinical data; and the Department of Biotechnology (DBT) for developing and defining regulatory guidelines. The agency follows the type II (abridged) review if the biosimilar has been approved by at least one recognised reference agency and waives the non-clinical studies if the product is already approved by more than one agency, including China and South Korea subject to positive review outcomes. The reference agencies defined for type II review model are EMA, MHRA, USFDA, TGA, and BRDD. In addition, the agency also carries out type III (full review) review but does not mention verification review model.

**SAHPRA -** The overall size of the agency is around 200 personnel. There is a total of five reviewers for biological applications with minimum qualification as shown in Table 2. The agency outsources CMC, non-clinical and clinical data evaluation to external evaluators and only allows a type III full dossier review.

**TITCK** – The agency follows the type III review model and takes advice from external assessors also for CMC, non-clinical and clinical review.

**COFEPRIS** - The biological division represents 1% of the overall size of the agency with a bachelor's degree as the minimum qualification for internal assessors. The agency relies more on external experts under both the committees, the SEPB (Sub-committee on evaluation of Biotech Products) and the NMC (New Molecule Committee) headed by COFEPRIS. The COFEPRIS is the only agency among these regulatory agencies of the emerging economies following the type I data assessment model relying on other reference agencies' evaluation including the EMA, USFDA, and TGA. In addition, the agency also conducts type III full dossier evaluation for biosimilars.

It is evident from Table 2 that SAHPRA, TITCK, CDSCO, and COFEPRIS agencies use the support of external assessors for review of application despite having an internal biologic division. This reflects a shortage of resources related to internal biologic reviewers. In addition, allocation of common assessors for biologic and non-biologic applications such as that practised by the Russian MoH may lead to suboptimal subject matter expertise. All the agencies follow “type III - Full review of the marketing authorisation application” data assessment model. In addition, CDSCO follows “type II - Abridged review” and COFEPRIS follows “type I – Verification review of marketing authorisation application.” This indicates that the reliance of these regulatory agencies of emerging economies on type I and type II models is less prevalent.

### Part II - Biosimilar Development Criteria

Establishing biosimilarity to the reference biologic product revolves around several steps starting from *in vitro* analytical testing and quality characterisation, non-clinical comparative pharmacology testing to toxicology, PK/PD studies, and clinical trials (clinical safety and efficacy) (14). Although it is evident, that the regulatory standards of these six emerging economies are mostly aligned and largely modelled on WHO guidelines (8), there is a lack of homogeneity in dossier requirements across these agencies posing a challenge to global development programmes. Such differences as presented by the regulatory agencies have been analysed and presented here.

#### Biosimilarity

All the six regulatory agencies of these emerging economies expect the sponsor to demonstrate biosimilarity of the proposed biosimilar product with the reference product. This includes proving satisfactory physicochemical and biological characterisation with *in vitro* non-clinical PK/PD studies and literature based clinical performance evaluation, additional *in vivo* safety data plus confirmatory clinical safety and efficacy trial. However, expectations for local or global clinical studies vary among the agencies. The ANVISA, SAHPRA, TITCK accept clinical studies performed in any country globally, while CDSCO and COFEPRIS mandate a local study. The Russian MoH accepts global studies as long as a trial includes Russian patients. In addition, extrapolation of indications is allowed subject to fulfilment of conditions defined by each agency.

Furthermore, these regulatory agencies of the emerging economies (except Russian MoH and TITCK) do not regulate interchangeability by law and allow a prescriber to decide based on a patient's need. However, in Russia, biosimilar products can be interchangeable with the reference product by law whereas in Turkey, the reimbursement institution authorises interchangeability.

#### Comparative Quality Characterisation

##### Reference Biologic Product (RBP) Selection

*Selection Criteria.* In response to questions on the RBP selection, the agencies mostly indicated mandatory requirements for locally authorised reference product (based on a full dossier submission including quality, safety, and efficacy) for comparability studies (Table 3).

**Table 3:** RBP selection criteria for six regulatory agencies of BRICS-TM.

**Regulatory agency**	**Selection criteria**	**Primary source of RBP**	**Alternate source of RBP**	**Use of RBP authorised in emerging countries**	**Criteria of RBP batches**	**Bridging study requirement**	**Data Sharing arrangement with other Regulatory agencies**
ANVISA	Approved based on full registration dossier with ANVISA	Locally authorised RBP	First innovator or biosimilar product authorised locally	Not accepted	Multiple batches of RBP with varied expiry dates	Not specified	EMA, USFDA, PMDA, MHRA
Russian MoH	Approved based on full registration dossier with Russian Federation	Locally authorised RBP	First innovator product authorised locally	Not accepted	Singe batch of RBP	Not specified	Not specified
CDSCO	Approved based on full registration dossier with CDSCO	Locally authorised RBP	ICH countries	Not accepted	Multiple batches (minimum 3 batches) of RBP with varied expiry dates	Not specified	EMA, USFDA
SAHPRA	Approved based on full registration dossier with SAHPRA	Locally authorised RBP	First innovator product authorised locally	Not accepted	Multiple batches of RBP with varied expiry dates (draft stage; but followed in practise)	Not specified	Not specified
TITCK^*^	Approved based on full registration dossier with TITCK	Globally authorised RBP	EMA, USFDA, BRDD, TGA, PMDA, MHRA, BfArM	Not accepted	Multiple batches of RBP with varied expiry dates (draft stage; but followed in practise)	Not specified	Not specified
COFEPRIS	Approved based on full registration dossier with COFEPRIS	Locally authorised RBP	EMA, USFDA, TGA, PMDA	Not accepted	Minimum 3 batches	Not specified	No data sharing arrangement and expects full dossier

* *The TITCK agency did not declare acceptable agencies, theoretically all countries are acceptable, extra data can be requested case by case; RBP, Reference Biological Product*.

*Primary and Alternate Source of RBP.* Flexibility in terms of sourcing the RBP from other ICH/reference countries exists in CDSCO, TITCK, and COFEPRIS, in the event of non–availability of locally authorised reference products. In addition, TITCK also accommodates use of a non-locally authorised RBP as well as locally sourced reference products for certain clinical safety studies (PK/PD study in human), non-clinical studies (*in vivo*), and development studies such as “quality target product profile” (QTPP) which is a summary of the quality characteristics of the respective biosimilar. These quality characteristics are essential to ensure that the finished product meets the required standard of quality.

*Use of RBP Authorised in Emerging Countries.* None of the agencies accept authorised reference products from other emerging countries, except CDSCO which may then only consider this in emergency situations such as the COVID-19 pandemic.

*Criteria of RBP Batches.* Unlike the Russian MoH and COFEPRIS, the regulatory agencies of Brazil, India, South Africa, and Turkey also mandate the use of multiple batches of RBP with varied expiry dates. However, ANVISA, has provisions for the changeover of RBP during development and comparability studies.

*Bridging Study Requirement.* All the six regulatory agencies of BRICS-TM has not specified the bridging study requirements.

*Data Sharing Arrangements.* ANVISA established data sharing arrangements with advanced regulatory agencies such as EMA, USFDA, PMDA, and MHRA. CDSCO also holds a data sharing agreement with EMA and USFDA. In contrast, COFEPRIS does not have a data sharing arrangement with other advanced regulatory agencies and expects a full dossier submission for products approved by a foreign agency (Table 3). Such arrangements of sharing of information about the product among the regulatory agencies would help the agency to understand if the RBP batch used for the development process has been made in the same facility or same process or same cell line and if the same information has been submitted to both the agencies. With evaluation of such shared data, the agency can waive the additional requirements on the RBP required for submission or waive the bridging studies. This type of data-sharing agreements would greatly decrease costs of biosimilar development.

The varied expectations for RBP sourcing from these agencies demonstrate the challenge in procuring multiple lots of RBP and the non-convergence in regulatory requirements, thereby limiting the opportunity for multi-country development.

##### Analytical Specification and Method

The similarity of physicochemical and biological properties of biosimilar and reference product is demonstrated using two or more orthogonal analytical methods (3). In keeping with this, the current assessment underlines the need for orthogonal methods for purity, impurity and contaminants characterisation as indicated by the responses from all the six emerging economies.

Furthermore, as specified clearly in WHO SBP guidelines (15), specifications for a SBP (Similar Biotherapeutic Product) will not be the same as for the RBP due to the difference in manufacturing process and analytical procedures followed by the manufacturer. Hence, specifications should be set based on the manufacturer's experience with the SBP (e.g., manufacturing history; assay capability; safety and efficacy profile of the product) and the experimental results obtained by testing and comparing the SBP and RBP. However, the regulatory agencies of these emerging economies' consideration for determining specifications and analytical methods for proposed biosimilar product varies across agencies.

The ANVISA and TITCK prefer analysis of multiple RBP lots with varied age along with the SBP. The COFEPRIS requires minimum of three batches of RBP. The Russian MoH predominantly expects specifications to be designed exactly the same as the RBP whereas CDSCO, SAHPRA, and COFEPRIS define specifications based on manufacturer's experience of the SBP and RBP, consistent with WHO guidelines.

##### Comparative Stability Studies

Four of the six regulatory agencies of these emerging economies (i.e., Brazil, Russia, South Africa, Mexico) indicated the need for comparative accelerated and stress stability studies, along with real time, real condition stability studies conducted in their respective climatic zone to support the shelf-life. CDSCO (India) does not require comparative studies and TITCK (Turkey) considers it only as supportive data for biosimilar development.

Comparative stability data is essential for “totality-of-evidence” to determine biosimilarity (16) and is an integral part of any biosimilarity assessment (17). As was evident from the responses, all the six regulatory agencies of these emerging economies are aligned with global standards in this aspect. However, CDSCO in practise, may consider an application even in the absence of side-by-side accelerated and stress stability studies though mandated as per the Guidance on Similar Biologics (18).

#### Non-clinical Studies

The six regulatory agencies of the emerging economies state that *in vitro* comparative functional assays such as biological assays, binding assays, and enzyme kinetics; *in vivo* pharmacokinetics, pharmacodynamics and immunogenicity studies; and *in vivo* comparative repeat dose toxicity studies are requisite for non-clinical studies. In addition, local tolerance studies and other toxicological studies are expected by CDSCO, TITCK, and COFEPRIS. Safety pharmacology studies are required by SAHPRA. In TITCK, the evaluation and acceptability are on case-by-case basis.

For *in vivo* studies, the Russian MoH advises the use of transgenic animal/ transplant models in a GLP setting while CDSCO suggests toxicity studies in rodent and non-rodent animals for proving statistical difference and advises to submit scientific justification for the choice of animal model. If a relevant non-rodent model is not available in India, then non-rodent studies can be waived by the Review Committee on Genetic manipulation (RCGM). The TITCK reported that the evaluation and acceptability of non-clinical studies is solely on a case-by-case basis in alignment with EU and ICH guidelines (19, 20). The rest of the six emerging agencies' responses were incomplete regarding the type and minimum sample size of each species for the study.

#### Clinical Studies

Table 4 presents the clinical trial requirements for biosimilar development in the six emerging economies.

**Table 4:** Clinical trial requirements for biosimilar development in emerging economies.

**Criteria**	**ANVISA (Brazil)**	**Russian MoH**	**CDSCO (India)**	**SAHPRA (South Africa)**	**TITCK (Turkey)**	**COFEPRIS (Mexico)**
**PK/PD studies (Phase I)**						
Combined PK/PD study	√	√	√	√	√	√
Requirement of immunogenicity studies	√ (Data can be obtained in PK/PD)	X	√ (Data can be obtained in PK/PD OR Phase III)	√	√	√(Data can be obtained in PKPD)
**Efficacy studies (Phase III)**						
***Study design***						
Randomised, parallel group, double-blind, adequately powered using efficacy endpoints	√	√	√	√	√	√
***Clinical study design acceptance***						
Equivalence design	√	√	√	√	√	√
Non-inferiority design	√	X	√	X	X	√
***Local clinical studies***	X	√	√	X	X	√
***Required in paediatric and elderly population***	X	√	√ (for extrapolated condition)	n/d	X	X
***Inclusion of third countries patients***	√	n/d	X	n/d	√ (if any genetic differences)	√

*n/d, Not defined; PK/PD, Pharmacokinetic/Pharmacodynamic; √, Required; X, Not required*.

Applicants need to submit PK/PD and clinical safety and efficacy studies data as part of a biosimilar application in all the six regulatory agencies of these emerging economies.

##### PK/PD

The PK/PD requirement in terms of design, endpoints, fingerprinting approach, and combining PK and PD studies are uniform across these emerging economies, closely aligned with the standards set by the EMA (20).

##### Immunogenicity

The responses from the agencies indicate the need for comparative immunogenicity as part of a biosimilar application, except for the Russian MoH. The CDSCO accepts that immunogenicity data can be obtained either from PK/PD or Phase III efficacy studies. Furthermore, all the agencies considered the extrapolation of immunogenicity studies to other indications, subject to the approved indications of the RBP. The expectations for such studies are defined in the Biosimilar Guidance 2016 in the case of CDSCO (18), however such clarity is yet to be defined by the other regulatory agencies of these emerging economies.

##### Comparative Clinical Efficacy Studies

*Clinical Study Design.* In general, all the regulatory agencies of these emerging economies studied expect a randomised, parallel group, double-blind, adequately powered clinical study using efficacy endpoints. Furthermore, ANVISA and COFEPRIS consent to both non-inferiority and equivalence design for clinical studies. The Russian MoH prefers an equivalence design, while CDSCO accept a non-inferiority design. In addition, the Russian MoH and CDSCO expect clinical comparability studies in paediatric and elderly populations in cases of extrapolated indications.

*Local Clinical Studies.* The ANVISA does not mandate performance of a local clinical study. However, for a global study, the agency mandates advice on regulatory expectations for clinical studies prior to protocol development, which is legally binding. Further, the foreign patient data is accepted by the agency as part of the biosimilar application if there are no foreseen genetic differences between the population studies and Brazilians. The TITCK has a similar requirement to that of ANVISA for acceptance of foreign patient data.

The Russian MoH requires local clinical studies for Phase III and mandates the inclusion of Russian patients when using global studies. Similarly, CDSCO requires local Phase III clinical trials in India. The sample size defined by CDSCO is a minimum of 100 patients in each arm. Usually, non-legally binding pre-submission advice is provided by the agency before the start of clinical trials. The agency does not accept foreign patient data as part of a biosimilar application.

As for COFEPRIS, the local clinical study requirement depends on the demonstration of comparability at CMC and non-clinical stages, as well as the robustness of the already performed clinical studies. The agency is open for inclusion of foreign patients in clinical efficacy studies for proving biosimilarity.

### Part III - Marketing Authorisation Approval Pathway

**Figure 1:**
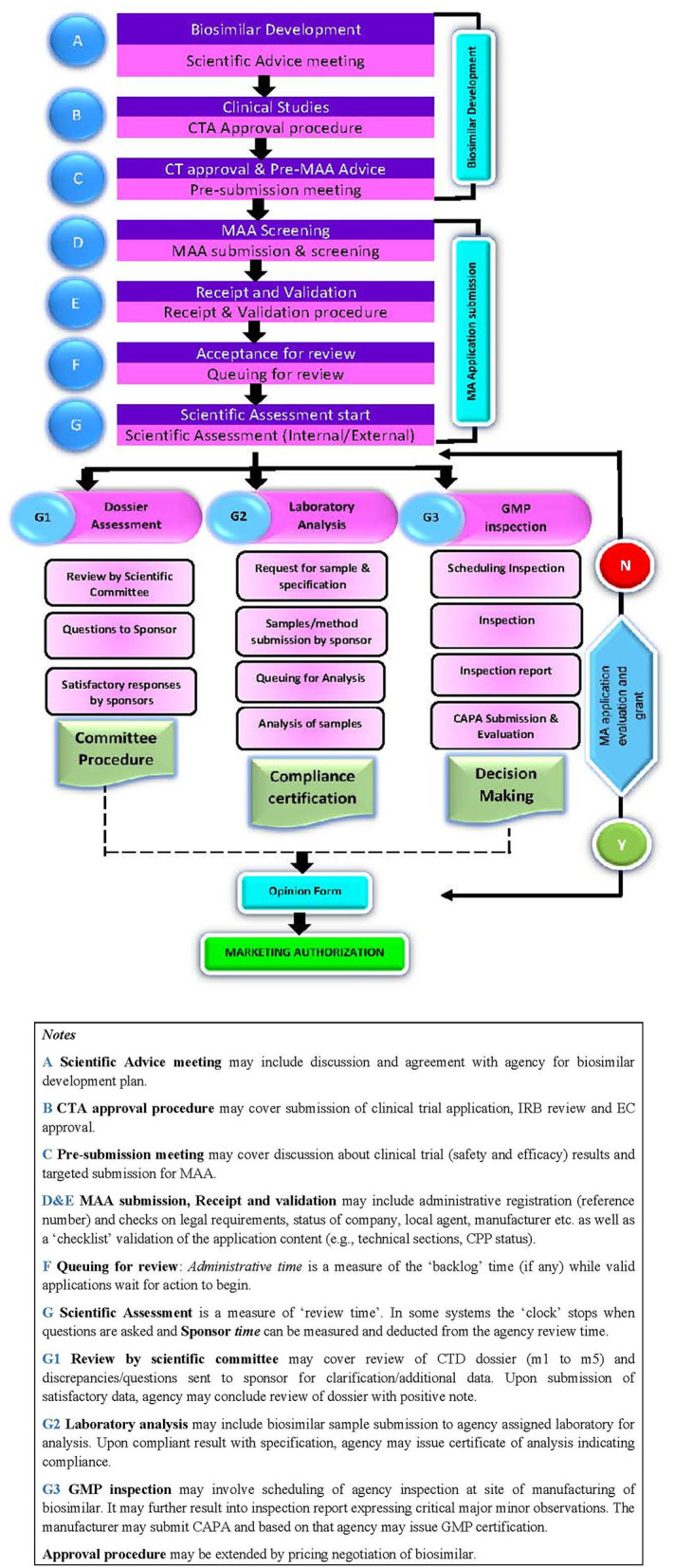
Marketing authorisation approval milestones.

The biosimilar application approval process includes the following steps: scientific advice, clinical trial application (CTA) approval process; and dossier review process including validation of application, queuing, scientific assessment, sample analysis, GMP certification, and product approval (Figure 1).

#### Scientific Advice

Throughout the development process of biosimilars, developers need the respective agency's advice. This can include; reference product selection and overall development strategy; evaluation and discussion post physicochemical and biological characterisation with *in vitro* non-clinical data; *in vivo* clinical data and justification of differences and clinical safety and efficacy trial protocol design and approval; and overall dossier content. The advanced agencies such as the USFDA (21) and the EMA (22) offer biosimilar developers formal meetings for scientific advice to perform appropriate tests and studies, so that no major objections regarding the design of the tests are likely to be raised during the review of the marketing authorisation application. This approach supports the timely and sound development of high-quality, effective and safe medicines for the benefit of patients and also helps to avoid patient studies that will not produce useful evidence.

Three of the six agencies (i.e., ANVISA, CDSCO, and SAHPRA) offer pre-submission advice for the biosimilar developers. The advice from ANVISA can be obtained through face-to-face meetings, electronic mails, or written correspondence whereas CDSCO and SAHPRA prefer face-to-face meetings. The expert advice received through such meetings is not legally binding on both parties, however, agencies do expect compliance to their comments during development of the biosimilar. The Russian MoH, TITCK, and COFEPRIS are yet to establish any formal meeting procedures.

The absence of scientific advisory meetings in TITCK has also been highlighted in an earlier study (23) where the importance of such interaction with agency has been emphasised. In Russia, face-to-face interaction between the government and a biosimilar manufacturer is not allowed and all regulatory communications must be carried out in writing (24).

#### Clinical Trial Application (CTA) Approval Process

The CTA is evaluated and approved by specific committees designated by the agencies such as: Coordenação de Pesquisa Clínica (ANVISA); Subject Expert Committee (CDSCO); and Clinical Trials Committee (SAHPRA). The Russian MoH and TITCK assign internal assessors to review the application. No clarity on this topic was received from COFEPRIS. An integral part of the CTA is the Ethics Committee (EC) approval letter, which is to be obtained from the Institutional Review Board of hospitals or institutions where the clinical trial is intended to be performed. The Russian MoH and COFEPRIS require an EC letter as part of the initial application, whereas the rest of the agencies are flexible and will accept such letter during the review process or post approval of the CTA.

All the regulatory agencies have varied timelines for CTA approval as shown in Table 5.

**Table 5:** Timelines for biosimilar review and approval process.

	**ANVISA**	**Russian MoH**	**CDSCO**	**SAHPRA**	**TITCK**	**COFEPRIS**
CTA review	90 days	45 days	90 days	<70 days^*^	30 days	45 days
Validation	Not applicable	5–15 days	No specific step	15 days	30 days	No information available
Queuing	60–180 days	No information available	14–56 days	<28 days	60–180 days	180–365 days
Scientific Committee review	30 days	30–90 days	No information available	60 days	No information available	90 days
Decision via committee meeting	Not applicable	30 days	Not applicable	≤ 240 days^#^	Not applicable	90 days
Issuance of Marketing Authorisation	<30 days	<30 days	90 days	<30 days	<30 days	90–180 days

* *There are cases where this turnaround time might be prolonged i.e., an unfamiliar investigational product which may be referred to external reviewers or other committees of SAHPRA for input for new applications*.

# *Registration within 240 days, may be earlier*.

*ANVISA, Russian MoH, SAHPRA, TITCK follows calendar days; CDSCO and COFEPRIS follows working days*.

#### Dossier Review and Approval Process

##### Dossier Content

The six regulatory agencies of the emerging economies accept electronic CTD dossiers as the format for marketing authorisation applications (MAA) for biosimilar products. The Certificate of Pharmaceutical Product (CPP) is a mandatory document as part of the initial dossier by the Russian MoH and CDSCO, for acceptance of the application by the agency. The ANVISA, COFEPRIS, and TITCK provide relaxation for the CPP submission before granting a marketing authorisation. In addition, TITCK also accepts any marketing authorisation certificate and published approvals from the relevant agencies' official websites.

Post submission by the sponsor, the product dossier passes through different stages such as screening against a checklist, acceptance for further review, queuing for review and scientific assessment resulting in approval or non-approval of the application by the agency.

##### Screening and Validation

As part of the screening or validation process, all the agencies verify applications against a standard checklist and request additional data (except CDSCO) if some documents are missing. In case of CDSCO, submission will not be uploaded on the SUGAM online portal if the dossier is inadequate. Further, all the information pertaining to “milestone” dates are recorded during the review process into an electronic tracking/recording system maintained by the agencies, i.e., DATAVISA (ANVISA), GRLS (Russian MoH), SUGAM (CDSCO). In case of SAHPRA and COFEPRIS, there is no specific system in place, whereas no information available from TITCK on the same.

##### Queuing

The queue time for dossiers awaiting review ranges from 4 weeks to 1 year as displayed in Table 5. All agencies, except the Russian MoH and COFEPRIS, confirmed that priority products including biosimilars are not required to be in a queue for review.

##### Scientific Assessment

Scientific assessment of biosimilar applications depends on the outcomes of the dossier review, sample analysis, and GMP certification.

For dossier review, CDSCO and SAHPRA use external assessors, however, there is no contractual agreement defining the timelines for review of the technical data. ANVISA and TITCK issue an emergency letter to sponsors in the case of a sudden unforeseen crisis as and when they review different sections of the dossier, while the rest of the agencies collate quality, safety, and efficacy deficiencies in one batch and send it to the applicant. The obligatory time for developers to respond to queries varies between 3 and 6 months and referred to as “clock stop.” Failure to meet the stipulated time, leads to rejection of the application with forfeiting of the fees with ANVISA, the Russia MoH, CDSCO, and COFEPRIS. The TITCK sends official letters for rejection to the sponsor, but the company can object to the same; however, there is no predetermined deadline in this aspect, while SAHPRA allows for extensions. Further, in case of a negative opinion from the scientific committee, CDSCO has provisions for sponsors to approach the technical committee and apex committee for their intervention and decision. The Russian MoH and ANVISA have no such additional provision and also there was no clarity received in this regard from COFEPRIS. The defined target timeline for scientific review by each of the regulatory agencies also varies as detailed in Table 5.

*Sample Analysis.* Most of the regulatory agencies of these emerging economies expressed a requirement of sample analysis at specified approved quality control laboratories as part of the dossier approval process. The ANVISA and SAHPRA rely only on technical documentation for biosimilar products and do not require sample analysis. The Russian MoH, TITCK, and CDSCO expect sponsors to submit samples along with analytical specifications and methods, reference/working standard and analytical columns. The CDSCO additionally require an analytical validation package. The maximum time to analyse samples is 110 calendar days as defined by FGBU (Russia) while no such deadlines are specified by other agencies.

*GMP Inspection.* These six regulatory agencies of emerging economies also mandate on-site GMP inspections for biological substances and biosimilar product manufacturing sites. Generally, each agency (except CDSCO) performs inspection during the dossier evaluation process, whereas CDSCO inspects site/s after completion of the dossier assessment. For TITCK, separate site GMP application is required, and the agency conducts inspection before scientific assessment of dossier, unless there are priority products. Also, CDSCO and SAHPRA accept GMP certification from reference agencies i.e., EMA (EU), BRDD (Canada), MHRA (UK), USFDA (USA) instead of on-site inspections. In addition, CDSCO accepts TGA (Australia) certification whereas COFEPRIS accepts EMA (EU), TGA (Australia), and USFDA (USA) certification. The TITCK does not accept foreign agencies' GMP inspections.

Across these emerging economies, the final decision maker on the marketing authorisation is the head of the agency.

##### Public Assessment Reports (PARs) and Approval Metrics

**Figure 2:**
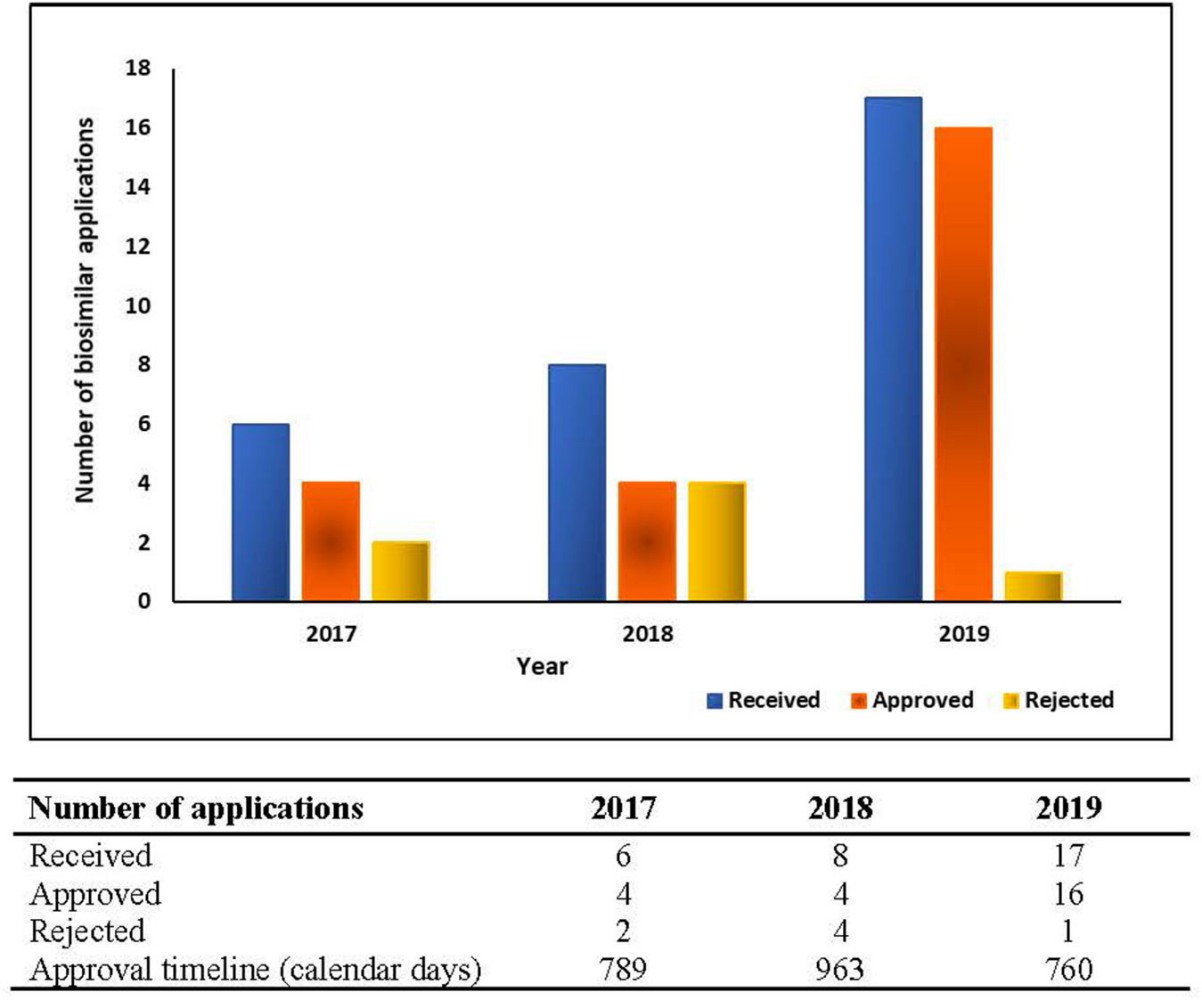
Biosimilar approval metrics of ANVISA – 2017–2019.

Except for ANVISA, the regulatory agencies of these emerging economies are yet to establish procedures for the issuance of a public assessment report or clarifying the basis for approval for the product. In such scenarios, measuring real approval timelines for biosimilars becomes arduous. The biosimilar approval metrics for the duration of 2017–2019 for ANVISA is presented in Figure 2.

## Discussion

Biosimilar products are complex molecules produced using highly complex manufacturing processes. Due to the complexity of the biosimilar products, regulatory requirements for analytical comparability, non-clinical, and clinical studies vary with the geographies (25), particularly in emerging economies like BRICS-TM, as evident from the secondary research (8). Furthermore, with multiple prospective manufacturers on the horizon, the need arises for a streamlined regulatory guideline in emerging economies that ensure biosimilarity, comparability, and interchangeability with respect to safety and efficacy of the product (25). Although substantial progress has been made in regulatory frameworks for chemical drugs, progress is less robust in developing countries, and implementing regulatory frameworks for biologic medicines, particularly biosimilar medicines (26). The recent studies reported in the literature suggest that the regulatory challenges in biosimilar space continue to be a topic of interest and deserves further debate. However, our study, in comparison to existing knowledge in the area, provides insight about TITCK (Turkey) and COFEPRIS (Mexico) agencies pertaining to biosimilar development challenges, in addition to the 20 countries included in the WHO survey reported by Kang et al. (27). The findings reposted by Garcia et al. (28) of Latin America are complemented by this study for challenges pertaining to biosimilar approval pathway. Furthermore, Sharma et al. (29) discuss global regulatory requirements on biosimilars and their difference amongst generics based on ophthalmic perspective while Cohen AD et al. (30) focuses on clinical practises specific for the treatment of psoriasis. However, our study reported here provides insight about biosimilar regulations irrespective of therapeutic areas.

Regarding the type of dossier assessment and allocation of resources for the dossier evaluation by the regulatory agencies, external evaluators are involved for review of applications by SAHPRA, TITCK, CDSCO, and COFEPRIS, while Russian MoH has common assessors for biologic and non-biologic applications. All the six emerging agencies follow “Type III - Full review of the marketing authorisation application” data assessment model. In addition, CDSCO follows “Type II - Abridged review” and COFEPRIS follows “Type I – Verification of marketing authorisation application”, with a clear indication of less prevalence of Type I and Type II review models among these countries. It has been commonly cited that building capacity and expertise in a national regulatory authority is a long-term process and quick resolutions lie in relying on information from other regulatory authorities or a shared or abridged review models (31). The study results reveal non-transparency and limited co-operation amongst the agencies for biosimilar medicinal product regulatory review. The outcome of this study may benefit these agencies by highlighting need for adopting shared review or reliance review models for scientific assessment of biosimilar applications. Evidence of shared review by SAHPRA (South Africa) with the national regulatory authorities of the member countries of the ZaZiBoNa work sharing initiative (32) such as Zambia, Zimbabwe, Botswana, Namibia offers an opportunity for an efficient and effective regulatory process for biosimilar evaluation in countries with limited resources. Brazil and Mexico are part of the PAHO (33) region, and Mexico uses the reliance model with authorities of regional reference, which includes the USA and Canada, thus using Type I review model for scientific assessment depending on the product. However, ANVISA, Brazil do not recognise any reference agencies for the dossier review and carry out full review (Type III). Although Brazil and Mexico are recognised as regional reference agencies in the America, the products authorised in these countries are neither recognised nor the data relied upon by other emerging economies within these regions.

The common expectations on demonstration of biosimilarity to the RBP across these emerging agencies includes satisfactory physicochemical and biological characterisation, *in vitro* non-clinical studies, additional *in vivo* safety data along with confirmatory clinical safety and efficacy data. While ANVISA, SAHPRA, TITCK accept global clinical studies, Russia MoH, CDSCO, and COFEPRIS mandates conduct of clinical efficacy trials in the local population. The non-acceptance of global clinical data and repetition of clinical studies mandatorily in local population by these regulatory agencies adds to unnecessary development costs (34). Such duplication of studies further is likely to impact the overall biosimilar development process and approval timelines in these countries (27).

With regards to the selection criteria for RBP and its procurement, these emerging agencies mandated locally authorised reference product (based on a full dossier including quality, safety, and efficacy) for comparability studies. While few agencies like CDSCO, TITCK, and COFEPRIS provide flexibilities for sourcing RBP from ICH/ reference countries, ANVISA, Russian MoH, and SAHPRA have stringent regulation on using only the locally licenced RBP. In Russia, a comparator product cannot be sourced from another regulatory jurisdiction since it is only allowed to use a reference comparator drug that has Russian marketing authorisation (35). Further, although most agencies expect multiple batches of RBP with varied expiry dates, the exact number of RBP batches required for comparability studies was not clearly defined by the agencies. Also, reference products authorised by other emerging countries are not accepted by these agencies, excepting in emergency situations, in case of CDSCO. There is seemingly a non-convergence in the regulatory requirements among these agencies with regards to RBP selection criteria. Acceptance of a non-locally licenced/sourced RBP by few countries and others that require a locally licenced reference product without any leverages, also demonstrates the challenge in procuring multiple lots of RBP, thereby posing a potential barrier for the biosimilar development process in these countries. It has been suggested by WHO, that exchange of information with other national regulatory authorities by accepting sourcing of non-locally licenced reference products, and avoiding unnecessary bridge studies (31) can circumvent such challenges in RBP sourcing.

In general, *in vitro* comparative functional assays are required by all the six emerging agencies along with *in vivo* pharmacokinetics, pharmacodynamics and immunogenicity studies. There is a mandatory requirement for *in vivo* repeat dose toxicity studies from all the countries; with TITCK evaluation in accordance with EU and ICH guidelines, on a case-by-case basis. Further, CDSCO expects non-clinical studies in rodent and non-rodent animal and Russian MoH in transgenic animal/transplant models to be conducted. However, there is no clarity on the type of study or species or other requirements from other agencies. The study clearly shows the lack of consistency in the regulations on non-clinical aspects from these countries. Also, such mandatory requirements for non-clinical studies demonstrates a lack of scientific approach toward the assessment of data indicating lack of full implementation of a “step-wise approach” for proving biosimilarity (36).

These six regulatory agencies of the emerging economies mandate PK/PD studies (regulations being similar to EMA), clinical safety and efficacy studies along with comparative immunogenicity data as part of a biosimilar application. However, the Russian MoH does not provide clarity on expectations on immunogenicity studies nor for extrapolation of immunogenicity data to other indications in most of the agencies. Further, as discussed earlier, the acceptance of clinical efficacy data from foreign patient data is not supported by all the agencies. Such a mandate on confirmatory clinical efficacy studies shows the agencies lack in science-based approach for review of dossier. It is apparent that the understanding by these agencies on the importance of comprehensive analytical comparability studies and the evaluation of comparability data for any structural and functional differences is inadequate. Further, non-recognition and non-acceptance of global data leading to duplication of studies, impacts on development costs and delays in approval of the biosimilar product (37). Emphasis on recognising data from other reference countries and the relevance of advanced analytical science to prove comparability in place of confirmatory clinical data has also been focussed by IGBA in their policy paper (36).

Scientific advice helps to ensure that developers perform the appropriate tests and studies, so that no major objections regarding the design of the tests are likely to be raised during the evaluation of the marketing authorisation application. This also helps avoid patients taking part in studies that will not produce useful evidence. Such pre-submission advice for biosimilar developers to get agencies' opinions on the biosimilar development process is only offered by a few of these emerging agencies like ANVISA, CDSCO, and SAHPRA. These advisory meetings are through face-to-face meetings, electronic mail or written responses. However, there are no set procedures for any formal meeting in the rest of these emerging agencies. The absence of a communication channel between the biosimilar developer and the national health authority greatly impacts the overall development process. With an increasing number of biosimilar developers across the globe, the scientific advice requests to developed agencies like EMA is expanding. EMA has also launched a pilot project in 2017 (38) for “tailored scientific advice” for the development path for biosimilar medicines, to test the added value and feasibility of the project. Implementation of scientific advisory meetings by these six regulatory agencies of emerging economies, similar to those by established regulatory authorities would support the potential manufacturers to have better clarity on the regional regulations and incorporate them in their global biosimilar development program.

The dossier content requirements for biosimilar Marketing Authorisation Application (MAA) are similar within these regulatory agencies of emerging economies. All these agencies accept electronic CTD and mandate CPP as part of the dossier, however the flexibility over time of the submission of such administrative documents (initial dossier or post approval of dossier) varies. Relaxation in terms of provision of other published approvals and authorisation documents in lieu of CPP by few agencies, exists. The dossier screening and validation process against a standard checklist is uniform across all the six emerging agencies, however the acceptance of the MAA with insufficient data differs with the agencies. The queueing time for dossier review varies from 4 weeks to 1 year, with almost all the agencies discounting the priority products (except the Russian MoH and COFEPRIS). The biosimilar application is considered for scientific assessment based on the outcome of the dossier review, sample analysis, and GMP certification. Although most of the agencies evaluate the dossier internally, a few opt for external evaluators for dossier evaluation. This is partly due to the full review of dossier (Type III data assessment model) by the agencies. Joint or shared review of the dossiers will ease the resource constraint or the dossier review process among these agencies (27). Further, such joint review can have a positive impact on the query response timelines, by allowing the sponsors to address the deficiencies in a single window rather than responding to the same query multiple times to different agencies. Such provisions might further minimise the number of dossier rejections within the agency, thereby allowing more biosimilars to penetrate into these emerging markets.

Despite the technical dossier, the requirement for samples by all these emerging agencies (except ANVISA and SAHPRA) along with reference standard/working standards for testing at qualified laboratories for the biosimilar approval process extends the overall biosimilar approval timelines. Additionally, each of these agencies mandate on-site GMP inspections for biological substances and biosimilar product manufacturing sites. Though inspection of the manufacturing site is essential for ensuring compliance to global manufacturing standards and assuring the quality of the product, individual or separate inspections by each of the emerging agencies leads to duplication. Instead, acceptance of reference agency GMP certification (including EU, PIC/S), as permitted by CDSCO, SAHPRA, and COFEPRIS will improve the process efficiency of the agencies (39). Also, collaboration, reliance or joint inspections among these regulatory agencies will minimise the resources and efforts required by developers, resulting in increased regulatory performance (39–41).

Based on the evaluation of the study results, following key outcomes from this study can be considered for an effective biosimilar development and approval process among these emerging agencies -

- This study emphasises the need to foster effective collaboration between regulators and developers in six emerging agencies in order to streamline the development strategies and approval pathways for biosimilar products.
- A formal approach to regular, appropriate, and tailored scientific advice from regulatory agencies to developers will help to align expectations on both sides and support step-by-step development, thereby reducing the need for certain studies i.e., *in vivo* non-clinical studies. This may also help to shorten the overall review and approval timeline.
- Significant challenges in sourcing RBP for comparative studies necessitates regulatory flexibility in norms for sourcing the comparator. Allowing RBP from other emerging countries will also facilitate the use of common biosimilar development programs.
- While appropriate resource allocation and upskilling of regulators needs to be considered, adoption of an alternative regulatory framework such as abridged review models might help in optimising the use of resources within the biosimilar departments of these six emerging agencies.

The biosimilar therapy in emerging economies is still in the infancy stage with little or no presence but expected to show strong growth (4) remains scope for improving transparency in the national regulatory frameworks and aligning regulatory standards among the emerging economies. In the light of the current global regulatory environment and the pandemic challenges, it was prudent for both regional and national regulatory authorities (NRAs) to re-evaluate regulatory requirement for development and approval of biosimilars taking into account the challenges faced by different stakeholders.

Although there were no remarkable changes in biosimilar guidelines in the six emerging economies between 2018 and 2020, there have been progress toward relaxing few guidelines with regards to conduct of clinical trials and GMP inspections. For instance, ANVISA, Brazil, has introduced certain relaxation of clinical trial procedure and allowed sponsors to modify or amend protocol without ANVISA's authorisation. In addition, if clinical study is related to COVID-19 then clinical trial consent can be obtained immediately upon formal submission of the protocol. As per the resolution of the Collegiate Board of ANVISA, RDC no 346/2020 of March 13th, ANVISA has adopted an alternative route for GMP certification of Active Pharmaceutical Ingredient (API), drugs and health products, based on remote inspection or reliance from other health authorities. If the manufacturer is accredited by PIC/s GMP certification, then ANVISA can process a faster GMP certification (42). RDC no 348/2020 of March 17th allows flexibility in evidence and prioritisation in analysis if the product has the therapeutic indication for treatment or prevention of the pandemic disease (43). Similarly, the Russian agency has taken step to have remote GMP inspections for foreign manufacturers (44).

The CDSCO has upgraded regulatory standards for clinical trials via the New Drug Clinical Trials Rule 2019 (45). In India, Schedule Y of Drugs and Cosmetics Act and Rules defined the requirement for clinical trials of new drugs and investigational new drugs for manufacturing and import prior to the New Drug Clinical Trial Rules (45) came into effect. The revised comprehensive NDCTR closes some of the gaps existing in Schedule Y in terms of number of subjects, nature and timing of non-clinical studies, content of the proposed protocol for performing clinical trials. As part of the first schedule, General Principles and Practises for Clinical Trial section (3) (2)(c) (iii), pertaining to new drugs approved outside India, the phase III study may need to be performed in India. It explicitly states that Phase III studies need to be carried out if scientifically and ethically justified to establish data for safety and efficacy of drugs in Indian patients. It further states that PK studies may be required by the Central Licencing Authority (CLA) in Indian patients. The CDSCO, India, has also developed rapid response framework for COVID-19 vaccines. Accordingly, the agency is open to considering pre-clinical or clinical data generated outside the country and shorten development requirements to reduce the time for approval (46). In addition, WHO GMP/ Certificate of Pharmaceutical Product (CoPP) extension of an additional 6 months has been provided and special permission has been granted to import drugs with <60% of remaining shelf life, up to October 2020. Similarly, the SAHPRA, South Africa, has issued policy documents for conducting clinical trials, based on the FDA's guidance on conduct of clinical trials of medicinal product during the COVID-19 pandemic (47).

Furthermore, The TITCK, Turkey, announced some flexibility due to COVID-19 such as postponing marketing authorisation certificate's annotation process, online stakeholder meetings regarding marketing authorisation activities, accepting CPP or similar certificate without apostille, readability test waiving until end of 2021, extension of GMP validity period to end of 2021 (48).

## Limitations of the Study

The study presented covers evaluation of responses obtained from 6 out of 7 (85.7%) regulatory agencies. While ANVISA, Russia MoH, CDSCO, SAHPRA, TITCK, and COFEPRIS responses were obtained, the multiple efforts to reach NMPA (China) either directly or via regulatory experts were unsuccessful. Though the non-participation of China in this study could be considered as a limitation, however the survey (even without China) encompasses a large, diverse and important segment of the world population and pharmaceutical market, so it should provide strategic information to pharma companies, as well as national regulatory authorities and international bodies. The response pertaining to biosimilar approval metrics i.e., applications received, applications screened and accepted for further review, biosimilars approved, biosimilars refused and average approval times was received only from ANVISA. The response from other agencies would have helped understand the process efficiency and provided benchmark in terms of basis of approval to biopharmaceutical companies.

## Conclusion

To conclude, many medical treatments and medicines now lay in Biotechnology, where understanding of the patient's physiology and cell make up is the key to treatment. Biological drugs bring that value in the treatment of many disabling and life-threatening chronic diseases, including inflammatory arthritis, certain types of cancer, diabetes, inflammatory bowel disease, Crohn's disease, psoriasis, and COVID-19. Biosimilars can help the public gain access to health through affordability, and that is where the need for the regulatory guidelines of biosimilars can contribute through harmonisation and simplification. The research undertaken presents an effort in that direction. This study has, for the first time, evaluated the regulatory requirement for approval and development of biosimilars in these six emerging economies and has identified a lack of alignment in certain areas that would benefit from standardisation. There remains scope for improving transparency in the national regulatory frameworks and aligning regulatory standards among these emerging economies. This would impact the overall review and approval process as well as enable a common development programme across these countries. Further, a future study could focus on developing proposals for an improved regulatory model for approval and development of biosimilars in these emerging economies. Integration of regulatory standards across emerging economies would also enable streamlined biosimilar development programmes and expedited licencing processes, thereby facilitating improvements in patient care and access to these life-saving medicines.

## Data Availability Statement

The original contributions presented in the study are included in the article/Supplementary Material, further inquiries can be directed to the corresponding author/s.

## Author Contributions

HR: designing of research methodology, questionnaire development, the study sample selection, online interviews, data collection, interpretation, and writing of the first draft of the manuscript. AS: review of the questionnaire, support in identifying appropriate participants for the study, and review of the first draft of the manuscript. GS: support in providing data from ANVISA, Brazil and review of the first draft of the manuscript and comments. CD: support in providing data for Russia and review of the first draft of the manuscript and comments. SP-T and CL-M: support in providing data for Mexico and review of the first draft of the manuscript and comments. SS: development of the concept, supervision of the study, review of the questionnaire, research coordination, interpretation of the results, and review of the first and final draft of the manuscript. All authors contributed to the article and approved the submitted version.

## Conflict of Interest

HR was employed by the company Metina PharmConsulting Pvt. Ltd. The remaining authors declare that the research was conducted in the absence of any commercial or financial relationships that could be construed as a potential conflict of interest.

## Publisher's Note

All claims expressed in this article are solely those of the authors and do not necessarily represent those of their affiliated organizations, or those of the publisher, the editors and the reviewers. Any product that may be evaluated in this article, or claim that may be made by its manufacturer, is not guaranteed or endorsed by the publisher.

## Abbreviations

ANVISA: Agência Nacional de Vigilância Sanitária
BDEA: Biosimilar Development Evaluation and Authorisation
BRDD: Biologic and Radiopharmaceutical Drugs Directorate
BRICS-TM: Brazil, Russia, India, China, South Africa, Turkey, Mexico
B.Sc.: Bachelor of Science
CDSCO: Central Drugs Standard Control Organisation
CECMED: The Centre for State Control on the Quality of Drugs
CEO: Chief Executive Officer
CLA: Central Licencing Authority
CIRS: Centre for Innovation in Regulatory Science
CMC: Chemistry Manufacturing and Control
COVID: Corona virus
COFEPRIS: Comisión Federal para la Protección contra Riesgos Sanitarios
COVID-19: Corona virus disease
CPP: Certificate of Pharmaceutical Product
CTA: Clinical Trial Application
DBT: Department of Biotechnology
EC: Ethics Committee
ECDA: European Chronic Disease Alliance
EMA: European Medicines Agency
EU: European Union
GDP: Gross Domestic Product
GMP: Good Manufacturing Practices
ICH: The International Council for Harmonisation of Technical Requirements for Pharmaceuticals for Human Use
IGBA: International Generic and Biosimilar Medicines Association
MAA: Marketing Authorisation Applications
MD: Doctor of Medicine
MHRA: Medicines and Healthcare products Regulatory Agency
MoH: Ministry of Health
NDCTR: New Drugs and Clinical Trial Rules
NMC: New Molecule Committee
NMPA: National Medical Products Administration
PAHO: Pan American Health Organisation
PAR: Public Assessment Report
PD: Pharmacodynamics
PharmD: Doctor of Pharmacy
PhD: Doctor of Philosophy
PIC/S: Pharmaceutical Inspection Co-operation Scheme
PK: Pharmacokinetic
PMDA: Pharmaceuticals and Medical Devices Agency
RBP: Reference Biological Product
RCGM: Review Committee on Genetic Manipulation
SAHPRA: South African Health Products Regulatory Agency
SBP: Similar Biotherapeutic Product
SEC: Subject Expert Committee
SEPB: Subcommittee on evaluation of biotech products
TGA: Therapeutic Goods Administration
TITCK: Türkiye Ilaçve Tibbi Cihaz Kurumu
US: United States
USFDA: United States Food and Drug Administration
WHO: World Health Organisation
ZaZiBoNa: Zambia, Zimbabwe, Botswana Namibia.
