# Supplementary material for: Current Regulatory Requirements for Biosimilars in Six Member Countries of BRICS-TM: Challenges and Opportunities
Source: Front Med (Lausanne). 2021 Sep 9;8:726660. doi: 10.3389/fmed.2021.726660 (PMC8458962; doi:10.3389/fmed.2021.726660)
Supplement: Supplementary file 1 [file Data_Sheet_1.PDF]

Regulatory Agency Perspective

Biosimilar Development, Evaluation and Authorisation Questionnaire

(BDEA)

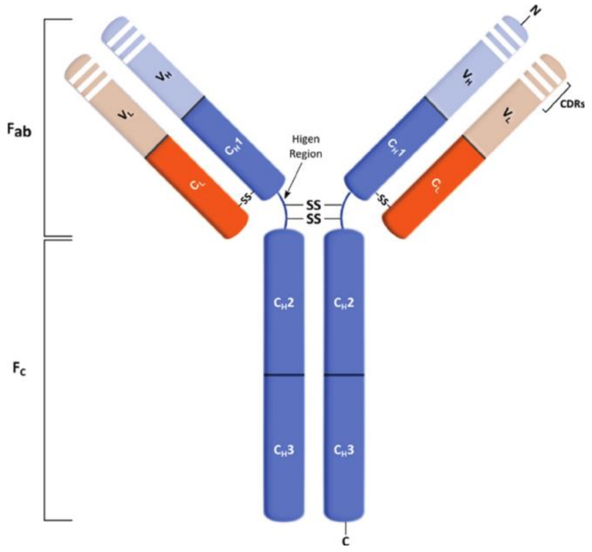

Copyright: Hasumati Rahalkar and Sam Salek, May 2020

CONFIDENTIAL

Regulatory Agency's views on Biosimilar Development and Marketing Authorization procedure in BRICS-TM markets

QUESTIONNAIRE

VERSION 013

MAY 2020

EC Approved Protocol Number: aLMS/PGR/UH/03332(1)

CONTENTS

Content	Page No.
Information for Participant ……………………………………………………….	1
Part I: Organization of the Agency………………………………………………….	4
Part II: Agency's views on biosimilar development criteria (biosimilarity criteria, reference biotherapeutic product selection, comparability criteria and content of biosimilar application) ………………………………………………………………	8
Part III: Marketing authorization approval pathway (Review of approval pathway, key milestones and target timelines for approval) ……………….………	25
Glossary and abbreviations…………………………………………………………	34

Confidentiality

All information collected from individual agencies will be kept strictly confidential. No data that will identify an individual agency will be reported or made available to any third party. External reports or presentations of the data will include only blinded results and any appropriate analytical interpretations.

Copyright: Hasumati Rahalkar and Sam Salek, May 2020

FOCUS OF THE STUDY

The study is intended, primarily, to document procedures and practices that relate to biological medicines that are the subject of **biosimilar** applications. Further, it also intends to document approval pathway, key milestones and target timelines for approval.

ABOUT THE QUESTIONNAIRE

The attached questionnaire is divided into three parts:

Part I: Organisation of the Agency: The **Introduction** to the questionnaire asks the respective regulatory authority to provide current information on its structure, organisation and resources pertaining to biosimilars.

Part II: Agency's views on biosimilar development criteria (biosimilarity principles, reference biotherapeutic product selection, comparability criteria and content of biosimilar application): This part of the questionnaire is established based on review of biosimilar development guidelines from EMA (EU), USFDA (USA), WHO, BGTD (Canada) and TGA (Australia) pertaining to quality, nonclinical and clinical comparability criteria against reference biotherapeutic product. It asks the respective regulatory authority to reveal requirements pertaining to biosimilar development for registration in their market.

Part III: Marketing authorization approval pathway (Review of approval pathway, key milestones and target timelines for approval): This part of the questionnaire is based on the general model giving a process map and milestones that has been developed from studying procedures followed in 'established' and 'emerging' regulatory agencies. It captures the main steps in the review and approval process and identifies key 'milestone' dates in the process for monitoring and analysing timelines. In addition, it also expects authority to define post-marketing compliances and approval matrix for biosimilar products.

OBJECTIVES

The objectives for each key point are as under:

Organisation of agency

- To identify administrative information i.e. size, scope, organisation structure, number of assessors, fees, funding, budget, etc. about agency pertaining to biosimilars

Agency's views on biosimilars development criteria

- To identify precise definition of biosimilarity principle as adapted by each agency
- To identify reference biotherapeutic product selection criteria for development of the biosimilar
- To identify comparability criteria in terms of characterisation, non-clinical and clinical studies for determining biosimilarity
- To understand agencies expectations on content of technical dossier for biosimilar marketing authorization application

Marketing Authorization Approval Pathway

- To identify biosimilar product approval pathway including key milestones and target timelines followed by each agency
- To identify biosimilars approval metrics in the country

OUTPUT

Participating agencies will receive a report from which they can compare their product development mandate, marketing authorization procedure and evaluation practices with those of peer agencies across the regions. This will include an analysis of gaps in biosimilar development requirements and evaluation practices.

Copyright: Hasumati Rahalkar and Sam Salek, May 2020

PART I: ORGANISATION OF THE AGENCY

Definition of Biosimilar:
A biosimilar is a biological medicinal product that contains a version of the active substance of an already authorized original biological medicinal product (reference medicinal product) in the EEA.

Reference: EMA: Guideline on similar biological medicinal products (CHMP/437/04 Rev 1)

1. Information on the Regulatory Agency

As background to the discussions about your agency, its practices and procedures it would be helpful to have the following basic information on its size and fee structure pertaining to biosimilar product:

Title of the Agency & division *responsible for the regulation of biosimilar products for human use*

____.

Size of agency

Please note that the following questions refer to the regulation of **biosimilar products for human use.**

1.1 Please provide information on staff numbers

- Total staff in the agency ☐
- Total staff in the biological division ☐
- Number of reviewers for **NEW** biological applications for marketing authorizations/ product licences ☐
- Number of reviewers for **BIOSIMILARS** applications for marketing authorizations/ product licences ☐

1.2 Please indicate the professional background and numbers of the ***technical*** *agency staff assigned to the review and assessment of biosimilar products.*

A. INTERNAL ASSESORS

Type of Assessors	*Total number of employees*	*Degree/Expertise (lowest to highest)*
• CMC		
• Non-clinical data		
• Clinical data		
• Microbiologists		
• Other Scientists		
• Project Managers		

B. EXTERNAL EVALUATORS

- Does agency appoint external evaluator? ☐ Yes ☐ No
- In which area, the maximum external evaluators are appointed so far?

☐CMC ☐Non-clinical ☐Clinical ☐Others (if any) __________________________

1.3 Biosimilar Committee

A. Does agency have biosimilar specific advisory committee?

☐Yes ☐No

If Yes, please provide title of the committee ____

B. How frequently advisory committee meets?

☐Once every month ☐Once in quarter ☐Once in half year ☐More than a month

1.4 In order, please provide title of all responsible division pertaining biosimilar approval.

____
____
____

Copyright: Hasumati Rahalkar and Sam Salek, May 2020

Fee structure

1.3 Are fees charged to sponsors for the review and assessment of applications for biological/biotechnological products for human use? ☐Yes ☐No		
If **Yes**, please provide the following information:		
Marketing Authorization Application fee for	***Local currency***	***US$ (rounded)***
☐New biological substance		
☐Established biological substance but non-biosimilar to reference medicinal product (Stand-alone development)		
☐Biosimilar product (with grant of interchangeable and substitution)		
☐Laboratory analysis fee		
☐GMP Inspection of manufacturing site		
☐GLP Inspection for analytical laboratory		
☐GCP Inspection of clinical site		
☐Clinical Trial Application (CTA) Approval		
☐Scientific advice and protocol assistance		
☐Advisory meeting (face-to-face)		
☐Post-marketing variations		
☐Annual maintenance fees		
☐Others (Please specify)		
Does the agency charge a fee for Scientific Advice via email/fax/corresponding letter (not seeking detailed review)?<br><br>☐**Yes** ☐**No**<br><br>If Yes, please provide fees applied to type of advice.		

Additional documentation

To assist us *to better understand your organisation, please provide copies of any* ***organisation charts*** *(including sample analysis division) that show the structure of the agency and its relationship to other regulatory bodies. It would also be very useful to have copies of any background papers that describe the* ***functions****,* ***remit*** *and* ***mission*** *of the agency. Also help us with the hard copy of* ***official fees regulation****.*

Copyright: Hasumati Rahalkar and Sam Salek, May 2020

2. Type of data assessment

Many agencies apply a different level of data assessment to different applications, according to the type of product and/or its regulatory status with other agencies. Three data assessment models for scientific review are described here.

Please indicate by checking the boxes below, which descriptions fit the model(s) used by your agency in the assessment of major applications.

Data Assessment Type I – Verification of Marketing Authorization Approval Application

This model is used to reduce duplication of effort by agreeing that the importing country will allow certain products to be marketed locally once they have been authorised by one or more recognised reference agencies, elsewhere. The main responsibility of the agency in the importing country is to 'verify' that the product intended for local sale has been duly registered as declared in the application and that the product characteristics (formulation, composition) and the prescribing information (use, dosage, precautions) for local marketing conforms to that agreed in the reference authorization(s).

☐ Data Assessment Type I followed ☐ Yes ☐ No

Specify for which Application Type I assessment is used

☐ All biosimilars approved by recognised reference agencies with approved dossier

☐ Used for selected applications (please specify with example of molecule)

____

____

____

☐ Others (Please specify including any restriction or conditions for approval)

____

____

____

Specify recognised reference agencies

☐ EMA (EU) ☐ USFDA (USA) ☐ BGTD (Canada)

☐ MHRA (UK) ☐ TGA (Australia) ☐ Others ______________________

Copyright: Hasumati Rahalkar and Sam Salek, May 2020

Data Assessment Type II – Abridged review of Marketing Authorization Approval Application

This model also conserves resources by not re-assessing scientific supporting data that has been reviewed and accepted elsewhere but includes an 'abridged' independent review of the product in terms of its use under local conditions. This might include a review of the biopharmaceutical (CMC) data in relation to climatic conditions and a benefit-risk assessment in relation to use in the local ethnic population, medical practice/culture and patterns of disease.

Approval by a recognised agency elsewhere is a pre-requisite before the local authorization can be granted.

☐ Data Assessment Type II followed ☐Yes ☐No

Specify for which Application Type II assessment is used

☐ All biosimilars approved by recognised reference agencies

☐ Used for selected applications (please specify with example of molecule)

____

____

____

☐Others (Please specify)

____

____

____

Specify recognized reference agencies

☐EMA (EU)	☐USFDA (USA)	☐BGTD (Canada)
☐MHRA (UK)	☐TGA (Australia)	☐Others (Please specify) ____________

Data Assessment Type III – Full review of Marketing Authorization Approval Application

In this model the agency has suitable resources, including access to appropriate internal and external experts, to carry out a 'full' review and evaluation of the supporting scientific data (quality, non-clinical, clinical) for a major application.

☐Used for all major applications

☐Used under the following conditions (please specify);

- ☐ Full review conducted but product must still be authorised by a reference agency prior to final authorization
- ☐ Full review results in the conditional approval/authorization for specific indication
- ☐ Full review results into labelling restriction
- ☐ Full review results into interchangeable and substitutable biosimilar product approval

If your agency has recognised 'reference agencies' (as in Types I and II) please provide the **copy of the regulation**

Copyright: Hasumati Rahalkar and Sam Salek, May 2020

PART II – AGENCY'S VIEW ON BIOSIMILAR DEVELOPMENT CRITERIA (BIOSIMILARITY CRITERIA, REFERENCE BIOTHERAPEUTIC PRODUCT SELECTION, COMPARABILITY CRITERIA AND CONTENT OF BIOSIMILAR MARKETING AUTHORIZATION APPLICATION)

This part of the questionnaire is based on the biosimilar development criteria for quality, non-clinical and clinical requirements from EMA (EU), WHO, USFDA (USA), BGTD (Canada), TGA (Australia) and emerging agencies guidelines.

3. Biosimilarity criteria

Demonstration of biosimilarity

3.1 As per your agency, which is the correct method for demonstrating biosimilarity with a reference product?

☐ Option 1: By proving satisfactory comparative physicochemical and biological characterization with *in-vitro* non-clinical (PKPD) study and literature based clinical performance evaluation

☐ Option 2: Option 1 plus additional *in-vivo* safety data

☐ Option 3: Option 1 & Option 2 plus confirmatory clinical safety and efficacy trial

☐ Others (Please specify) ____

If confirmatory clinical trial is mandatory, does it require to be performed in your country?

☐ Yes ☐ Not mandatory, it can be performed in well-established markets only

☐ Not mandatory, it can be performed anywhere globally

☐ Not mandatory, it can be performed in BRICS-TM markets

3.2 Which of the below change in biosimilar product as compare to reference product fall into biosimilarity scope as per your agency?

☐ Excipients	☐ Change in cell line
☐ Change in manufacturing process	☐ Difference in container closure (primary)
☐ Change in few test parameters	☐ Change in impurity profile (higher/lower)
☐ Varied storage conditions	☐ Decreased/Increased shelf life
☐ Reduced toxicity	☐ Lower immunogenicity
☐ Number and type of adverse reaction	☐ Change in the glycosylation pattern
☐ Improved efficacy	☐ Others (if any)

____

____

Posology and route of administration

3.3 Does the agency expect the posology and route/s of administration of proposed biosimilar to be the same as for the reference biological product?

☐ Yes ☐ No ☐ Others (if any) _______________

Pharmaceutical form

3.4 Does the agency expected pharmaceutical form of the proposed biosimilar to be the same as that of the reference biological product?

☐ Yes ☐ No ☐ Others (if any) _______________

Strength/Biological activity

3.5 Does agency expect the strength/biological activity of the proposed biosimilar to be the same as that/those for the reference biological product?

☐ Yes ☐ No ☐ Others (if any) _______________

Copyright: Hasumati Rahalkar and Sam Salek, May 2020

Formulation (including excipients)

3.6 Does agency expect the formulation (including excipients) of the proposed biosimilar to be the same as that/those for the reference biological product?

☐ Yes ☐ No ☐ Others (if any) _______________

Pack Presentation

3.7 Does the agency allow different presentation/s of the proposed biosimilar than that/those of the reference biological product?

☐ Yes ☐ No ☐ Others (if any) _______________

Extrapolation of indications

3.8 Under which circumstances, does the agency allow extrapolation to other indications for biosimilar application?

☐ With scientific justifications based on study in sensitive condition
☐ Post-marketing surveillance
☐ Clinical safety and efficacy in one condition
☐ Same mechanism of action for all conditions
☐ Justification based on published and pharmacopeial proof
☐ Justification based on extrapolation of indications of reference biological product
☐ Others (if any)

____

____

3.9 Does the agency allow extrapolation of indications in pediatric populations?

☐ Yes ☐ No
☐ Others (if any)

____

____

Biosimilarity post approval

3.10 Does agency expect to prove biosimilarity for changes performed post marketing authorization of biosimilar (i.e manufacturing changes)?

☐ No need to prove biosimilarity ☐ Yes, need to prove biosimilarity
☐ Others (if any)

____

____

If yes, which of below comparative studies are expected by agency?

☐ Option 1: Physico-chemical and biological characterization
☐ Option 2: Option 1 plus *in-vitro* non-clinical study
☐ Option 3: Option 1, Option 2 and clinical safety and efficacy study
☐ Option 4: Confirmatory clinical trial

If yes, which of below comparison is expected by agency?

☐ Comparative study against original reference product
☐ Comparative study against previously manufactured biosimilar by the same company
☐ Comparative study against original product and also previously manufactured biosimilar by the company
☐ Others, please specify ____

____

Copyright: Hasumati Rahalkar and Sam Salek, May 2020

Interchangeability: Interchangeability refers to the possibility of exchanging one medicine for another medicine that is expected to have the same clinical effect at agency level.

3.11 Is interchangeability allowed by law in your country?

☐ Yes (specify law) ___________________

☐ No ☐ Law under progress

☐ Others (if any)

____

____

3.12 Please indicate the type of interchangeability accepted within your agency.

☐ Reference biological product interchangeable to biosimilar

☐ Biosimilar interchangeable to reference biological product

☐ Biosimilar interchangeable to another equivalent biosimilar

☐ Others (if any)

____

____

____

Switching: It is when the prescriber decides to exchange one medicine for another medicine with the same therapeutic intent.

3.13 Is switching allowed in patients in your country?

☐ Naive ☐ Non-naive

☐ In-treatment

☐ Others (if any)

____

____

____

3.14 As per law who decides on the switching of products in patients in your country?

☐ Physicians ☐ Patients

☐ Pharmacists

☐ Others (if any)

____

____

____

Substitutions: It is the practice of dispensing one medicine instead of another equivalent and interchangeable medicine at pharmacy level without consulting the prescriber.

3.15 Is substitutions between products allowed at pharmacy level in your country?

☐ Yes ☐ No

☐ Allowed, if manufacturer is the same as for the product

☐ Others (if any)

____

____

Copyright: Hasumati Rahalkar and Sam Salek, May 2020

Pediatric research

3.16 Does the agency expect to have a pediatric investigation plan to be submitted as part of biosimilar application with your agency?

☐ Yes ☐ No

☐ Others (if any) ________________

Please specify if pediatric waiver or deferral submission is available with your agency.

____

____

Biosimilars naming : Under biosimilar naming, the INN name designated for each product will be a proper name (reflecting certain scientific characteristics of the product, such as chemical structure and pharmacological properties) that is a combination of the core name and a distinguishing suffix that is devoid of any meaning.

3.17 Is there a specific naming system mandatory for biosimilar products in your country?

☐ Yes ☐ No ☐ Under progress

☐ Others (if any) ________________

If yes, please indicate the criteria of specific naming/suffix to INN

____

____

____

Please indicate specific naming criteria for interchangeable products with your country.

____

____

____

Labelling

3.18 Is there specific guideline published by your country for labelling of biosimilar products by your agency?

☐ Yes ☐ No

☐ Under progress

☐ Others (if any)

____

____

If yes, does guideline specifies differences in labelling of reference biological product, biosimilar product and interchangeable product?

____

____

Does the authority demand to have specific statements for biosimilar and for interchangeable products?

☐ Yes ☐ No

If yes, please specify statement over here

____

____

____

Copyright: Hasumati Rahalkar and Sam Salek, May 2020

4. Choice of Reference Product Selection

4.1 What are the criteria to select a reference product for developing a biosimilar product?

- ☐ Reference product must be approved in your country.
- ☐ Reference product approval is based on full registration dossier like quality, safety and Efficacy in your country.
- ☐ Reference product must be marketed for substantial period in your country.
- ☐ Reference product must have sufficient volume of marketed use in your country.
- ☐ Others (if any)

____

____

4.2 If the reference biologic product is not authorized in your country, is a foreign approved product acceptable?

☐ Yes ☐ No

☐ Others (if any)

____

____

If yes, which agency approved product is acceptable with your agency?

☐ EMA (EU) ☐ USFDA (USA)

☐ BGTD (Canada) ☐ TGA (Australia)

☐ PMDA (Japan) ☐ MHRA (UK)

☐ BfArM (Germany)

☐ Other (Please specify)

____

____

4.3 Is reference product from other emerging markets acceptable to your agency?

☐ BRICS-TM market

☐ Others (if any)

____

____

4.4 Does your agency expect identity of the original reference product?

☐ Brand name ☐ Pharmaceutical form

☐ Manufacturing site details ☐ Location/Place from where reference product sourced

☐ Expiration detail and other labelling requirements

☐ Others (if any) ____

____

4.5 Does your agency allow to change reference product during development and comparability study?

☐ Yes ☐ No

☐ Others (please specify) ____

____

4.6 Does your agency expect to have a full dossier access for products approved by a foreign agency?

☐ Yes ☐ No

☐ Others (if any)

____

____

Copyright: Hasumati Rahalkar and Sam Salek, May 2020

4.7 Does your agency have data sharing arrangement with below agency?<br>☐ EMA (EU) ☐ USFDA (USA) ☐ BGTD (Canada)<br>☐ TGA (Australia) ☐ PMDA (Japan) ☐ MHRA (UK)<br>☐ BfArM (Germany)<br>☐ Other (Please specify)<br>____<br>____
4.8 Does your agency allow to use a foreign reference biologic product in comparability studies, even though a local approved reference biologic product is available?<br>☐ Yes ☐ No<br>☐ Others (Please specify)<br>____<br>____<br><br>*If yes, in which studies are foreign reference product can be used instead of locally approved product?*<br>☐ Clinical safety study ☐ Non-clinical study (*In-vivo*)<br>☐ PKPD study in human ☐ During development in laboratory for QTPP finalization<br>☐ Anyone of above study (Product Specific)<br>☐ Others (Please specify)<br>____<br>____<br><br>*Please provide details if any bridging study between original reference product and locally approved product is required.*<br>____<br>____
4.9 Does your agency agree to use another biosimilar product instead of original reference product as reference product for full comparability study?<br>☐ Yes ☐ No<br>☐ Others (if any)<br>____<br>____
4.10 Does your agency mandates the usage of multiple batches of reference biologic product with varied expiry dates during development of biosimilar product?<br>☐ Yes ☐ No<br>☐ Others (if any)<br>____<br>____<br><br>*If No, can only one batch of reference product be utilised throughout development?*<br>☐ Yes ☐ No

5. Comparative quality (characterization) attributes

5.1 What kind of in-vitro in-vivo assay tests are required to justify biological activity of proposed biosimilar product*?*<br>☐ Binding ☐ Enzymatic<br>☐ Cell-based ☐ Function-based

Copyright: Hasumati Rahalkar and Sam Salek, May 2020

5.2 What are the basic parameters for comparative characterization of proposed biosimilars' mAb structure, expected by your agency?

☐ Primary and higher order structure (class, subclass kappa and lambda chain determination)

☐ Amino acid sequencing (confirmation of variability at N- and C- terminal)

☐ Groups and bridges (free sulfhydryl group and disulphide bridge determination with analysis of integrity and mismatch of bridges).

☐ Carbohydrate content and structure (with oligosaccharide pattern confirmation)

☐ Glycosylation site(s) presence or absence on Fc region and heavy chain with occupancy Confirmation (including pattern and site occupancy).

☐ Glycan structure characterisation

☐ Others (if any) please specify

____

____

5.3 Which of the below immunological properties are essential for comparative characterisation of proposed biosimilar product by your agency?

☐ Antigen binding assay (including affinity, avidity and immunoreactivity)

☐ Cytotoxic evaluation for unintended target tissue (CDC and ADCC activity)

☐ Cross-reactivity determination

☐ CDR (Complementarity-determining region) identification

☐ Epitope characterization (including biochemical identification and determination of epitope with bearing molecule)

☐ Complementary ability evaluation (Evaluation of binding and activation and/or effector functions)

☐ Others (if any) please specify

____

____

5.4 Does your agency expect product effector functions to be characterised as part of biosimilar product development?

☐ ADCC (Antibody-dependent cellular cytotoxicity) analysis

☐ Complement binding ability ☐ Cytotoxic properties (e.g. apoptosis)

☐ Fc- gamma receptor binding activity and neonatal receptor binding ability

☐ Others (if any)

____

____

5.5 Does your agency expect to utilize orthogonal methods for purity, impurity and contaminants characterisation (orthogonal methods are method used in addition to primary method for specificity and selectivity of protein)?

☐ Yes ☐ No

☐ Others (if any)

____

5.6 As part of physicochemical comparative characterisation of proposed biosimilar product, does your agency expect below analysis as part of biosimilar marketing authorization application?

☐ Purity ☐ Contaminants

☐ Structural heterogeneity ☐ Multimers, aggregates and particulate matter

☐ Impurity profile and process-related impurities

☐ Others (if any)

____

____

Copyright: Hasumati Rahalkar and Sam Salek, May 2020

5.7 Does the agency permit the usage of cell lines that are different for a proposed biosimilar product than was used for the reference biological product?<br>☐ Yes ☐ No<br>☐ Recommendation (if any)<br>____<br>____
5.8 Does your agency expects Hybridoma cell line details (origin & characteristics data) as part of dossier?<br>☐ Yes ☐ No<br>☐ Others (please specify)<br>____<br>____
5.9 Which of the below cell line information (detailed) is expected by agency as part of biosimilar application?<br>☐ Immunization of animal ☐ Isolation of spleen cells<br>☐ Cultivation of myeloma cells ☐ Fusion of myeloma and B cells<br>☐ Separation of cell lines<br>☐ Others (if any) ______________________________<br>____
5.10 Does the agency expect detailed information about immortalization of the cell line for proposed biosimilar product?<br>☐ Yes ☐ No<br>☐ Others (if any) ______________________________<br>____
5.11 Which of the below parameter is considered by the agency for determining the quantity of the proposed biosimilar product?<br>☐ Biological activity ☐ Expression system<br>☐ Others (if any) ______________________________<br>____
5.12 Which of these below parameters expected by agency for validation batches to develop evidence for registration dossier?<br>*Number of batches*<br>☐ 1 pilot scale batch ☐ 3 pilot scale batches<br>☐ 3 commercial scale batches ☐ Others (please specify) ________________<br>*Minimum expected batch size*<br>☐ 1/10th of production capacity ☐ Equivalent to commercial production<br>☐ Minimum possible batch size ☐ Others (please specify) ________________
5.13 What are the agency's view points on determining analytical specifications and test selections for proposed biosimilar product?<br>☐ Specifications same as reference biologic product<br>☐ Specification based on manufacturer's experience on SBP and RBP<br>☐ Based on RBP's number and age of lots, time of testing and time of quality attributes<br>☐ Others (if any)<br>____<br>____

Copyright: Hasumati Rahalkar and Sam Salek, May 2020

5.14 Does the agency expect applicant to submit comparative accelerated and stress stability studies with reference biological product for development stage?

☐ Yes ☐ No

☐ Others (if any)

If yes, does the study require for drug substance and drug product both?

____

____

5.15 Does agency expects stability studies required both in the intended and a representative container closure system, as part of development data?

☐ Yes ☐ No

☐ Others (if any)

____

____

5.16 Which of the below stability time points data is expected by agency as part of marketing authorization application?

☐ 3 months long-term and 3 month accelerated stability data

☐ 6 months long-term and 6 month accelerated stability data

☐ 12 months long-term and 6 month accelerated stability data

☐ Full shelf life data

Which of the below storage conditions stability data is expected by agency as part of Marketing Authorization Application?

☐ Zone II (25°C & 60% RH) ☐ Zone IVa (30°C & 65% RH)

☐ Zone IVb (30°C & 75% RH)

5.17 Does the agency expect to prove compatibility between 1) biological substance and excipients and 2) primary packaging material and biological substance/excipient, if it is different than reference biological product?

☐ Yes ☐ No

☐ Others (if any) ____

____

5.18 Does the agency accept biosimilar application which is manufactured using platform manufacturing technology (production strategy based on similar manufacturing processes i.e. pre-defined process, cell culture and purification process)?

☐ Yes ☐ No

☐ Others (if any) ____

____

If yes, what would be expectation of documentation for final application?

☐ Validation studies of final manufacturing process and site

☐ The approval of similar products (manufacturing processes) in other countries

☐ Others (if any) ____________________

If no, kindly explain reason?

____

____

Copyright: Hasumati Rahalkar and Sam Salek, May 2020

6. Comparative non-clinical data

In-vitro studies

6.1 For comparative in-vitro studies, which of the below binding and functional tests are mandatory as per your agency?

☐ Binding target antigen(s) assay

☐ Binding assays with Fc gamma (FcγRI, FcγRII, FcγRIII) receptors, FcRn and complement (C1q)

☐ Fab- associated functions (soluble ligand neutralization, activation or blockade of receptor)

☐ Fc-associated functions (ADCC, ADCP, CDC, complement activation)

☐ Others (if any) ______________________________

____

In-vivo studies

6.2 Which of the following comparative in-vivo studies are mandatory as part of proposed biosimilar application with your agency?

☐ PK (Pharmacokinetics)

☐ PD (Pharmacodynamics)

☐ Repeated-dose toxicity

☐ Unspecific toxicity

☐ Immunogenicity

☐ Safety pharmacology

☐ Reproduction toxicology

☐ Carcinogenicity

☐ Local tolerance

☐ Others (if any) ______________________________

____

It would be great, if you will help us with **guidelines prints for *in-vitro* and *in-vivo* studies.**

6.3 Which of the studies are mandated to be performed locally at GLP centre located anywhere globally? Please guide on type and minimum sample size of species for each study to be performed locally.

Study	Type of animal species	Sample size
1. PKPD *in-vivo*		
2. Toxicity		
• Single dose toxicity study		
• Repeated-dose toxicity study		
• Unspecific toxicity		
• Reproduction toxicology		
• Carcinogenicity		
3. Immunogenicity		
• Comparative safety pharmacology		
• Local tolerance		
Others, if any		

Copyright: Hasumati Rahalkar and Sam Salek, May 2020

7. Comparative clinical data

Pharmacokinetics (PK)

7.1 Please indicate which therapeutic dose is essential for PK studies with your agency?

☐ Lowest therapeutic dose ☐ Highest therapeutic dose

☐ Others (if any) ____

____

Kindly request you to explain the reason for above dose decision by your agency.

____

____

7.2 Does your agency have any preference for route of administration for PK studies?

☐ Subcutaneous route ☐ Intravenous route

☐ Others (if any) ____

Kindly request you to explain the reason for above route of administration decision

____

____

7.3 Please indicate sampling points expected in PK studies as per your agency's guideline?

A. Single-dose study
   - ☐ Till last quantifiable concentration
   - ☐ Others (if any) ____

B. Multi-dose study
   - ☐ First dose and steady state
   - ☐ Others (if any) ____

7.4 Please indicate PK study design expected by your agency?

A. Short half-life products
   - ☐ Single dose cross-over with late elimination phase
   - ☐ Single-dose parallel with late elimination phase
   - ☐ Others (if any) ____

B. Long half-life products
   - ☐ Single dose cross-over with late elimination phase
   - ☐ Single-dose parallel with late elimination phase
   - ☐ Others (if any) ____

7.5 Please indicate primary and secondary endpoints for PK study expected by your agency?

A. Primary endpoints
   - ☐ Single dose AUC (0-inf), for subcutaneous administration C_{max} as co-primary parameter
   - ☐ Multiple dose- truncated AUC after the first administration until the second administration $AUC_{(0-t)}$, AUC over a dosage interval at steady state (AUC_{τ})
   - ☐ Others (if any) ____

B. Secondary endpoints
   - ☐ Single dose C_{max}, T_{max}, V_{ss}, $t_{1/2}$ ☐ Multiple dose C_{max} & C_{trough}
   - ☐ Others (if any)____

Copyright: Hasumati Rahalkar and Sam Salek, May 2020

Pharmacodynamics (PD)

7.6 Does your agency accept combined PKPD studies for a proposed biosimilar application?

☐ Yes ☐ No

☐ Others (if any) ____

____

If the answer is No, please explain the reason.

____

____

7.7 Does your agency accept clinical biosimilar comparability established based on fingerprinting (PK studies supported by non-surrogate PD/biomarkers) approach?

☐ Yes ☐ No

☐ Others (if any) ____

____

If the answer is No, please explain the reason.

____

____

Clinical efficacy

7.8 For clinical efficacy studies, do you agree with randomized, parallel group, double-blind, adequately powered using efficacy endpoints (in absence of surrogate markers) study type?

☐ Yes ☐ No ☐ Others (if any) ______________________

7.9 Which of the following design is highly acceptable with your agency?

☐ Equivalence design ☐ Non-inferiority design

☐ Comparability Phase-III clinical design ☐ Superiority design

☐ Others (if any) ______________________

7.10 Please indicate, if a clinical efficacy study is required in pediatric and elderly population for proving comparability of proposed biosimilar application by your agency?

☐ Yes ☐ No ☐ Others (if any) ______________________

7.11 Does your agency mandate local clinical data generation?

☐ Yes ☐ No

If Yes, what is the minimum sample size ______________________________

7.12 Does your agency allow inclusion of third countries patients in clinical efficacy studies for proving biosimilarity?

☐ Yes ☐ No ☐ Others (if any) ______________________

Clinical safety

7.13 Does your agency expect applicant to submit comparative immunogenicity studies as part of biosimilar application (pre-approval process)?

☐ Yes ☐ No ☐ Others (if any) ______________________

If yes, can this data be obtained in PKPD studies?

☐ Yes ☐ No

Does your agency allow to extrapolate immunogenicity to other indications?

☐ Yes ☐ No ☐ Others (if any) ______________________

If no, kindly indicate reasons ______________________________________

Does your agency have any guidelines for immunogenicity study?

☐ Yes ☐ No ☐ Under progress

If no, please advise which other agency guideline to be reference

____

____

Copyright: Hasumati Rahalkar and Sam Salek, May 2020

7.14 Does your agency mandate local clinical data generation?

☐Yes ☐No

If Yes, what is the minimum sample size ______________________________

7.15 Does your agency specify regulatory expectations for clinical studies prior to protocol development?

☐Yes ☐No

If Yes, is this advise legally binding for both agency and developer?

☐Yes ☐No

7.16 Which of the studies are mandated to be performed locally at specific centre accredited by your agency and please guide on minimum sample size for each study?

**Clinical Study**	**Sample size**
PK in human	
PD in human	
Combined PKPD	
Clinical Efficacy	
Clinical Safety	
Combined clinical safety and efficacy	

Pharmacovigilance and Risk management plan

7.17 Does your agency have electronic reporting system for adverse reaction?

☐Yes ☐No ☐Others (if any) ____________________

7.18 In the absence of specific biosimilar naming guideline, how does different biosimilars adverse reaction reported?

☐By biosimilar active substance ☐By biosimilar active substance and sponsor name

☐Others (if any) ____

____

7.19 Does your agency expect applicant to submit PV planning and risk management plan along with biosimilar application?

☐Yes ☐No ☐Others (if any) ____________________

If yes, can this data be obtained in PKPD studies?

☐Yes ☐No

8. Batch Analysis prior to market release

8.1 Does your agency mandatorily perform each batch analysis before releasing into market?

☐Yes ☐No

If yes, how many batches are required to be analysed?

☐Initial 3 batches ☐ Annually 1 batch ☐ Each batch

☐Others (Please specify)

____

____

What would be timeline for batch sample analysis?

☐15 days ☐1 month

☐2 months ☐3 months

Copyright: Hasumati Rahalkar and Sam Salek, May 2020

8.2 As part of batch analysis, what documents are expected by agency?<br>☐ Certificate of Analysis ☐ Analytical specification and method of analysis<br>☐ Batch Sample ☐ Reference/Working standard<br>Fees (Please specify the fees to be paid) ____________________<br>Any other (Please specify) ____<br>____
8.3 Does agency/ assigned analytical laboratory would withdraw samples from the batch?<br>☐ Yes ☐ No
8.4 Does agency have internal equipped analytical laboratory for biosimilar testing?<br>☐ Yes ☐ No<br>If No, is there any contractual agreement with third party laboratory?<br>☐ Yes ☐ No

9. GxP

GMP Inspection

9.1 Is manufacturing site GMP certification mandatory by your agency, then which pathway will be followed by your agency<br>☐ *On-site GMP inspection* ☐ *Document based GMP verification*<br>*Does applicant need to submit separate application for site GMP inspection?*<br>☐ Yes ☐ No
9.2 When does an agency schedule a GMP inspection?<br>☐ Before scientific assessment of dossier ☐ After scientific assessment of dossier
9.3 What is the deadline for manufacturer to submit CAPA against inspection report?<br>☐ 30 days ☐ 60 days ☐ Others (if any) ____________
9.4 Does your agency recognize foreign agencies GMP inspection?<br>☐ Yes ☐ No<br>Which agencies inspections are recognized?<br>☐ EMA (EU) ☐ Canada (BGTD) ☐ UK (MHRA)<br>☐ Australia (TGA) ☐ USA (USFDA)<br>☐ Any other agencies (Please specify) ______________________________<br>*Please provide copy of* ***GMP Inspection guideline***

9.5 Does your agency issues GMP certification based on documents review considering reference agencies GMP certificate?<br>☐ Yes ☐ No

Copyright: Hasumati Rahalkar and Sam Salek, May 2020

If Yes, tickmark documents reviewed

☐ GMP certificate ☐ Most recent inspection report
☐ Regulatory inspection list ☐ Regulatory action details
☐ Site master file ☐ GMP agreement
☐ Release for supply procedure
☐ Any others

____
____

Fees for desktop GMP certification ______________________

9.6 When does agency GMP inspection is initiated?

☐ Before product dossier submission ☐ After product dossier submission
☐ Anytime between product dossier submission and approval
☐ Other (If any, please specify)

____
____

9.7 What kind of GMP certificate are issued by agency?

☐ Product specific GMP certificate ☐ Full manufacturing line GMP certificate

9.8 What would be duration of GMP certificate?

☐ 5 years ☐ 3 years ☐ 1 years ☐ Others

9.9 What would be minimum timeframe required for renewal of GMP application submission?

☐ 3 months ☐ 6 months ☐ 1 year

GLP

9.10 Does your agency need all non-toxicological studies to be performed at GLP center?

☐ Yes ☐ No

GCP

9.11 Does agency accepts clinical data generated at internationally approved GCP center located globally?

☐ Yes ☐ No

If No, then will your agency mandatorily perform GCP inspection of the site?

☐ Yes ☐ No

9.12 Please specify acceptable international agency's GCP accreditation.

____
____

9.13 Will this inspection be pertaining to specific clinical studies or in general?

____

9.14 What would be duration of GCP certificate?

☐ 5 years ☐ 3 years ☐ 1 years ☐ Others ______________

9.15 What would be minimum timeframe required for renewal of GCP application submission?

☐ 3 months ☐ 6 months ☐ 1 year

Copyright: Hasumati Rahalkar and Sam Salek, May 2020

10. Foreign manufacturer

10.1 What, if any, are the additional data points/ studies/ requirements needed for a foreign Biosimilar developer over and above locally developed/ manufactured Biosimilar?

☐ *Registration Fees* ☐ *Non-Clinical*
☐ *Clinical* ☐ *Sample Analysis*
☐ *GMP certification* ☐ *GCP certification*
☐ *Others* ____

____

11. Content of Biosimilar Marketing Authorization Application

11.1 Does your agency need local registration holder for submission of dossier?

☐ Yes ☐ No

11.2 Is it mandatory to obtain tradename before submission of dossier to the agency?

☐ Yes ☐ No

11.3 In the absence of CPP documents, which information would agency accepts as part of dossier submission?

____
____
____

For those applications where prior authorization with other agency is essential please answer the following questions about the Certificate of a Pharmaceutical Product (CPP)

Is the inclusion of a CPP an absolute requirement before accepting the application as valid?

☐ Yes ☐ No ☐ For some applications (please specify) ______________________

If Yes must the CPP be legalized by an Embassy or Consulate?

☐ Yes ☐ No

If No, please indicate which of the following apply

- A CPP must be provided before the authorization is issued
  ☐ Yes ☐ No
- Other evidence of authorization by other countries is accepted in place of the CPP (e.g., copy of authorization, Internet reference)
  ☐ Yes ☐ No

11.4 Which type of dossier is acceptable to your agency?

☐ Paper CTD ☐ CTD in PDF files, electronic
☐ Software generated eCTD dossier

11.5 What is the ***representative sample quantity*** *to be submitted along with dossier?*

____
____

11.6 Does your agency need patent declaration /certification as part of dossier submission?

☐ Yes ☐ No

11.7 Which of the following legalized documents required by your agency as part of dossier?

☐ Manufacturing license ☐ GMP certificate
☐ Certificate of Pharmaceutical Product

Copyright: Hasumati Rahalkar and Sam Salek, May 2020

11.8 Does your agency need labelling in local language?

☐ Yes ☐ No

11.9 Does your agency require a CPP equivalent document as part of the review?

☐ With application ☐ Before authorization ☐ Not essential

11.10 Does your agency accept other documentation from the authorising agencies accepted as evidence of registration?

☐ Letter of authorization ☐ Copy of full authorization

☐ Internet evidence

☐ Other (Please specify)

____

____

11.11 Does your agency allow Priority/fast track application?

☐ Yes ☐ No

If Yes, please explain with an example

____

____

*Please provide guideline for **fast track application procedure**.*

11.12 Does your agency have published monoclonal antibodies (mAbs) biosimilar/product-class specific development guideline?

☐ Yes ☐ No

☐ Under progress (please specify tentative timeline for publication)

☐ Others (if any)

____

____

11.13 Does agency accept biosimilar application even when the original product patent is valid?

☐ Yes ☐ No

If yes, how many months before patent expiry biosimilar application can be filed?

☐ 6 months ☐ 12 months ☐ 24 months ☐ 36 months

Copyright: Hasumati Rahalkar and Sam Salek, May 2020

PART III: MARKETING AUTHORIZATION APPROVAL PATHWAY (REVIEW OF APPROVAL PATHWAY, KEY MILESTONES AND TARGET TIMELINE FOR APPROVAL)

Model Marketing Authorization Approval Pathway and Milestones

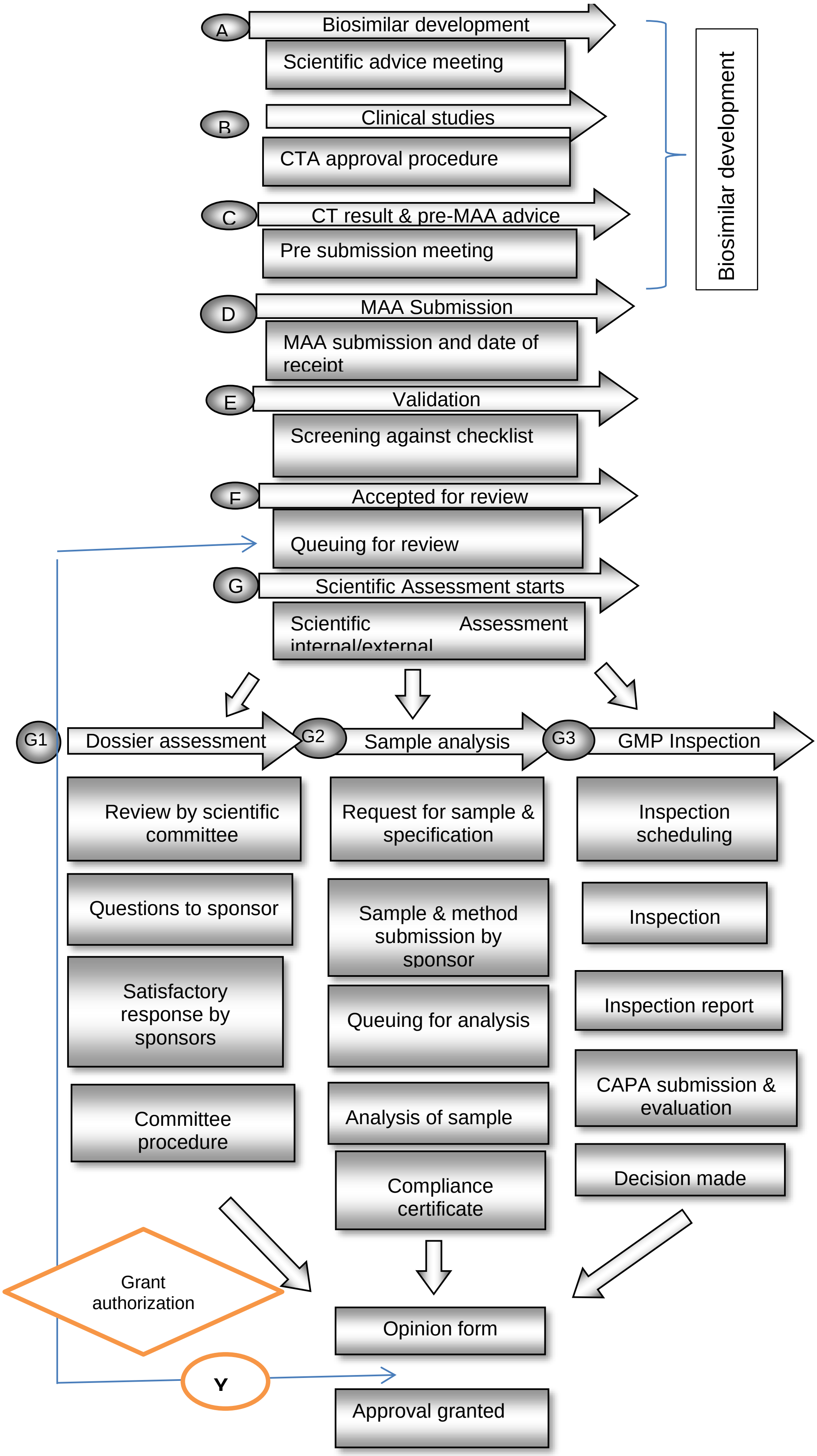

Notes

A Scientific advice meeting may include discussion and agreement with agency for biosimilar development plan.

B CTA approval procedure may cover submission of clinical trial application, IRB review and EC approval.

C Pre submission meeting may cover discussion about clinical trial (safety and efficacy) results and targeted submission for MAA.

D MAA submission

E Validation may include checks on legal requirements, status of company, local agent, manufacturer etc. as well as a 'checklist' validation of the application content (e.g., technical sections, CPP status).

F Queuing for review: *Administrative time 1* is a measure of the 'backlog' time (if any) while valid applications wait for action to begin.

G Scientific Assessment is a measure of 'review time'. In some systems the 'clock' stops when questions are asked and **Sponsor *time*** can be measured and deducted from the agency review time.

G (1) Review by scientific committee may cover review of CTD dossier (m1 to m5) and discrepancies/questions sent to sponsor for clarification/additional data. Upon submission of satisfactory data, agency may conclude review of dossier with positive note.

G (2) Laboratory analysis may include biosimilar sample submission to agency assigned laboratory for analysis. Upon compliant result with specification, agency may issue certificate of analysis indicating compliance.

G (3) Product specific GMP inspection may involve scheduling of agency inspection at site of manufacturing of biosimilar. It may further result into inspection report expressing critical major minor observations. The manufacturer may submit CAPA and based on that agency may issue GMP certification.

Approval procedure may be extended by pricing negotiation of biosimilar.

Copyright: Hasumati Rahalkar and Sam Salek, May 2020

12. General Information

12.1 When information is given on target or actual times please indicate here whether these are counted in:

☐ Calendar days ☐ Working days

12.2 When 'milestone' dates are recorded during the review process is the information entered into an electronic tracking/recording system?

☐ Yes**,** system in current use ☐ No, system in development

☐ No, a manual system will be used for the foreseeable future

13. Scientific advice

13.1 Does your agency offer scientific advice to sponsor for biosimilar development and further MAA submission?

☐ Yes ☐ No

☐ Others (if any) ________________________________

If yes, whether this advice is in which form?

☐ Face-to-face meetings ☐ Paper/E-mail based written response

13.2 At which of the below stages, agency offer scientific advice?

☐ Reference product selection and overall development strategy

☐ Evaluation and discussion post physicochemical and biological characterisation with *in-vitro* non-clinical data

☐ *In-vivo* clinical data and justification of differences

☐ Clinical safety and efficacy trial protocol design and approval

☐ Others (if any) ____________________

Please specify parameters which are out of scope for agency advice?

____

13.3 Is there a formal procedure i.e. letter of intent to be submitted to agency for obtaining meeting schedule?

☐ Yes ☐ No

Please provide a template for the meeting request along with detailed data requirement i.e. briefing package, followed by agency.

____

13.4 What would be official timeline for the meeting?

☐ 15 days ☐ 30 day ☐ 45 days

Others (if any) ____

13.5 Does agency expect fees from sponsor for providing advice?

☐ Yes ☐ No

If yes, please specify fees applicable for 2020.

____

13.6 Is the scientific advice provided through meeting, legally binding?

☐ Yes ☐ No

Please provide rationale behind above decision.

____

Copyright: Hasumati Rahalkar and Sam Salek, May 2020

13.7 How many meetings are entitled to sponsor throughout one biosimilar development?<br>☐1-3 years ☐4-5 years ☐5-10 years<br>If other, specify number of meetings<br>____

14. CTA approval procedure

14.1 Does your agency have specific committee for evaluation and grant of clinical trial application?<br>☐Yes ☐No<br>*If yes, please specify the name of the committee.*____________________<br>*A. How many times in a month, do the committee members meet for evaluation of CTA?*<br>☐Once a week ☐Twice a month<br>☐Specify (if different) ____________________<br>*B. Are all the committee members internal evaluators?*<br>☐Yes ☐No<br>*If No, what is the percentage of external evaluators for evaluation of CTA?*<br>____
14.2 What is the timeline defined by your agency for review of CTA?<br>☐30 days ☐45 days<br>☐Specify (if different) ______________________________
14.3 Can EC approval letter be submitted during review of CTA application to agency, instead of being done sequentially?<br>☐Yes ☐No<br>*Please provide checklist or guideline for clinical trial application*
14.4 Please provide standard fees for CTA approval by your agency?<br>____
14.5 In case of major change in clinical trial protocol post-approval, what is the timeline followed by your agency for approval of such changes?<br>Please specify timeline ______________________________

15. Pre-submission meeting

15.1 Does your agency allow pre-submission meeting with sponsor to define legal basis of application based on clinical results?<br>☐Yes ☐No

16. MAA submission

16.1 Are there any formal requirements before an application is submitted, for example, notification of intent to submit, assignment of registration code etc.<br>☐Yes ☐No

Copyright: Hasumati Rahalkar and Sam Salek, May 2020

Validation

16.2 Is the date of application receipt formally recorded?	☐Yes	☐No
If Yes, is specific file number issued?	☐Yes	☐No

Acceptance for review/refusal to file

16.3 Is the date of application acceptance formally recorded? ☐Yes ☐No

16.4 What happens if the application is incomplete?

☐**Refusal to file:** New application must be made

☐**File pending:** A request for the missing data is sent to the applicant

What is the time limit for the applicant to reply?

Target time for validation

16.5 Is there a target validation time? ☐Yes ☐No

Please specify time in days: ____

17. Queuing/backlog

17.1 Which of the following applies to the queuing system for new applications?

☐Held in queue before validation starts ☐Held in queue after validation

17.2 What is the current queue time (approximately)?

☐Less than 2 weeks ☐2-8 weeks

☐2-6 months ☐6 months-1 year

☐More than 1 year

17.3 Are priority products taken out of turn in the queuing system?

☐Yes**,** always ☐Yes, sometimes

☐No, all applications await their turn

Please specify which products:

____

17.4 Does the Agency regard the backlog of applications as a problem?

☐Yes ☐No

If Yes, how is this being addressed?

____

____

17 Scientific Assessment

Initiation of scientific review

18.1 Is the start of the Scientific Assessment formally recorded with official letter?

☐Yes ☐No

18.2 Is the scientific data separated into three sections (quality, safety, and efficacy) for review?

☐Yes ☐No

18.3 In what order are the different sections assessed?

☐ In parallel ☐In sequence

Copyright: Hasumati Rahalkar and Sam Salek, May 2020

If in sequence, please give order
____

*18.4 Who carries out the **primary** scientific assessment?*

☐ Agency technical staff ☐ Sent to outside experts
☐ Different procedure for different sections
Please describe the process: ____

Use of outside experts

18.5 If outside experts are used for the assessment of scientific data, please complete the following:
Number of experts on the agency's list or panel ______________

Main responsibility:
☐ To provide a detailed assessment report and recommendation
☐ To provide a clinical opinion on the product
☐ To provide advice to the agency staff on specific technical issues
☐ Other (specify)

Is there a contractual agreement on working within deadline set by the agency?
☐ Yes ☐ No

Interaction with the sponsor

18.6 How are questions send to the sponsor?
☐ As they arise during the assessment ☐ Collected into a single batch

18.7 When are batched questions sent to the sponsor?
☐ After the initial assessment but before reporting to scientific committee
☐ Not until the scientific committee has given its advice
☐ Before and after reference to the scientific committee

18.8 Does the scientific review cease while questions are being processed by the sponsor ('clock stop')
☐ Yes ☐ No

18.9 Is the sponsor given a time limit to reply?
☐ Yes ☐ No

If **Yes**, what time is allowed?	

18.10 What happens, if sponsor fails to respond within allocated time?
☐ Application is rejected with forfeiture of fees
☐ Application is rejected with possibility to reuse fees with fresh file
☐ Fresh application to be submitted with query response
Please specify the % of fees for forfeited ______________________

Meetings

18.11 Can the Sponsor hold meetings with the agency staff to discuss questions and queries that arise during the assessment ☐ Yes ☐ No

If Yes, what conditions and restrictions (if any) are applied?

1. Request formal meeting ☐ Yes ☐ No
2. Require scientific argument to be provided beforehand ☐ Yes ☐ No
3. Guideline is available to describe this procedure ☐ Yes ☐ No

Copyright: Hasumati Rahalkar and Sam Salek, May 2020

Review by Scientific Committee

18.12 Is a Committee of Experts (internal and/or external) used in the review process?

☐ Yes ☐ No

18.13 If Yes, at which stage in the review?

☐ Responsible for the whole assessment of the dossier from the start of the review
☐ Integrated into the agency's own internal/external scientific review procedure
☐ Consulted after the agency has reviewed and reported on the scientific data
☐ Other (specify) ____________________

18.14 Are the dates at the start and end of the Committee Review recorded?	☐ Yes	☐ No
18.15 Is the agency mandated to follow the Committee recommendation?	☐ Yes	☐ No
18.16 Is there a time limit for the Committee Procedure?	☐ Yes	☐ No
If Yes, please give the target		
If No, what is the time range (e.g., 1-3 months)		

18.17 Is there an additional step in the scientific review process, after the Committee has given its opinion?

☐ Yes ☐ No

If Yes, please describe briefly the work carried out at this stage (e.g. final report and agency opinion)

____

Target for scientific review

18.18 Is a target time set for the scientific review		☐ Yes	☐ No
If Yes**,** please give target			

19 Sample analysis

19.1 Which institute is responsible for performing analysis on sample?
Please provide the name

____

19.2 What is the timeline for completing sample analysis post receipt of sample?

☐ 10 days ☐ 20 days
☐ Specify (if other)

Please specify the standard fees for sample analysis ____________________

19.3 Does your agency expect sponsors to provide analytical accessories?

☐ Analytical specification and method ☐ Analytical validation package
☐ Analytical column ☐ Reference/working standards

____

Please specify average quantity of samples required ____________________

Copyright: Hasumati Rahalkar and Sam Salek, May 2020

20 Decision on the Application

Responsibility for the authorization decision

20.1 Who makes the decision that a marketing authorization can be granted?

☐ The Scientific Committee ☐ The Head of the Agency

☐ The Minister of Health ☐ Others

Other Criteria to be met

20.2 Is the authorization dependent on a pricing agreement ☐ Yes ☐ No

If Yes, when are the pricing negotiation started?

☐ At the start of the scientific review ☐ After the end of the scientific review

☐ After the start but before the end of the scientific review

20.3 Is the issue of the authorization dependent on sample analysis ☐ Yes ☐ No

If Yes, when is the analytical work started?

☐ In parallel with the scientific review ☐ At the end of the scientific review

☐ After the start but before the end of the scientific review

20.4 Is there a separate negotiation of the product labeling/product information after the scientific opinion is given but before the approval is issued? ☐ Yes ☐ No

Comments____

____

20.5 Please specify any other legal/administrative matters that must be finalised before the approval can be issued

____

____

Approval procedure

20.6 Is the Sponsor informed of a positive scientific opinion before the authorization is issued? ☐ Yes ☐ No

20.7 Approximately how long does it take from receiving a positive scientific opinion to issuing an approval?

☐ Less than a month ☐ 1-3 months ☐ 3-6 months ☐ Over 6 months

Comment:

21 Biosimilars Approval Metrics

It would be very helpful to have the following information on processing times for marketing authorizations that have been determined in the three years 2017, 2018, 2019.

Applications received

**Type**	***Number of applications received in each year***			**Current backlog**
	**2017**	**2018**	**2019**	
**Biosimilars**				

Copyright: Hasumati Rahalkar and Sam Salek, May 2020

Applications screened and accepted for further review

**Type**	***Number of applications determined in each year***		
	**2017**	**2018**	**2019**
**Biosimilars approved**			
**Biosimilars refused**			

Average approval times

**Type**	***Time from receipt of application to issue of approval***		
	**2017**	**2018**	**2019**
**Biosimilars**			

Target for approval times

21.1 Is a target time set for the overall approval process? ☐ Yes ☐ No

If Yes, please give target time ☐

Please comment on the actual review times in relation to the authority's target time.

___

___

Please provide copy of ***list of biosimilars*** *approved by your agency.*

___

___

Availability of public assessment report or equivalent document

21.2 Does the agency issues public assessment report or equivalent document indicating the basis of product approval in country?

☐ Yes ☐ No

If No, what are the ways by which industry can obtain approval summary?

___

___

22. Concluding Observations

The purpose of the following three questions is to try to identify the Agency's challenges and upcoming plan for making biological product available to meet patients' needs.

22.1 List three major challenges faced by agency in assigning marketing authorization for biosimilar product in your countries.
1.
2.
3.
22.2 List three initiatives which your agency is planning to implement in next two years' time to guide applicants in developing biosimilar product for your countries.
1.
2.
3.
22.3 *List three factors that act as barriers to making biosimilar product available in a timely manner through the regulatory process*
1.
2.
3.

Copyright: Hasumati Rahalkar and Sam Salek, May 2020

List of documents submitted along with Questionnaire by the Agency		
**Sr. No.**	**Document name**	**Yes/No**
1	Organisation chart	
2	Official fees regulation	
3	Reference agencies circular	
4	Stability study guidelines	
5	Biosimilar related guidelines	
6	Non-clinical study guideline	
7	Clinical study guideline	
8	GMP certificate guideline	
9	Fast track application circular/guideline	
10	CTA guideline	
11	List of biosimilars approved by agency	

Please provide additional documents listed above together with the completed questionnaire directly to Hasumati Rahalkar (Email id: hr17abj@herts.ac.uk).

____

____

Contact details

Name: Mrs Hasumati Rahalkar<br>Institute Name: University of Hertfordshire<br>Phone: +91 9820113613<br>Email id: hr17abj@herts.ac.uk	Name: Professor Sam Salek<br>Institute Name: University of Hertfordshire<br>Phone: +44 7763574022<br>email: m.s.salek@herts.ac.uk/sssalek52@gmail.com

Thank you for completing this Questionnaire

Please sign and date:

Name:	Position:
Agency name:	Signature:
Date:	Email address:

Copyright: Hasumati Rahalkar and Sam Salek, May 2020

Glossary and Abbreviations

**Characterization**	This characterisation should include the determination of physicochemical and immunochemical properties, biological activity, purity, impurities and quantity of the monoclonal antibody.
**Comparability exercise**	The activities, including study design, conduct of studies, and evaluation of data, that are designed to investigate whether the products are comparable.
**CMC**	Chemistry, manufacturing and controls
**Common Technical Document (CTD) format**	Common technical document (CTD) as outlined in the ICH guideline M4 (Organisation of the common technical document for the registration of pharmaceuticals for human use; M4).
**Marketing Authorization Application (MAA)**	Authorization application submitted to a regulatory authority to launch a drug product on the market to which the application has been submitted.
**Validation of a dossier**	The process whereby the authority verifies that all parts of the submitted dossier are present and complete and suitable to be assessed as part of the assessment and registration process.
**Marketing Authorization Approval**	Authorization issued by a regulatory to launch a drug product on the market.
**PK studies**	Comparative pharmacokinetic (PK) studies designed to demonstrate similar PK profile of the biosimilar and the reference medicinal product with regard to key PK parameters. The design of a PK study depends on various factors, including clinical context, safety, PK characteristics of the reference product (target-mediated disposition, linear or non-linear PK, time-dependency, half-life, etc.)
**PD studies**	The branch of pharmacology concerned with the effects of drugs and the mechanism of their action.
**Clinical safety study**	It is the pharmacological science relating to the collection, detection, assessment, monitoring and prevention of adverse effects.
**Clinical efficacy study**	The ability to produce a desired effect i.e. appropriate pharmacological activity for a specified indication.
**Extrapolation to other indication**	Extrapolation is the approval of a biosimilar for use in an indication held by the originator biologic not directly studied in a comparative clinical trial with the biosimilar.
**Reference Biotherapeutic Product**	The reference medicinal product must be a medicinal product authorised in the EEA, on the basis of a complete dossier in accordance with the provisions of Article 8 of Directive 2001/83/EC, as amended.

Copyright: Hasumati Rahalkar and Sam Salek, May 2020

**Pharmacovigilance (PV)**	Pharmacovigilance is the science and activities relating to the detection, assessment, understanding and prevention of adverse effects or any other medicine-related problem.
**Risk Management Plan**	Risk Management Plan is a document that describes the current knowledge about the safety and efficacy of a medicinal product.
**GCP**	Good Clinical Practice
**ICH**	International Conference on Harmonisation
**Joint review**	The whole dossier is reviewed by each authority and the outcome is discussed before a decision is taken.
**Peer review**	Peer review means an additional evaluation of an original assessment carried out by an independent person or committee. Peer review can occur either during assessment of a dossier, or at sign-off.
**Sponsor**	A company, person, organisation or institution that takes responsibility for initiating, managing or financing a clinical study.

Copyright: Hasumati Rahalkar and Sam Salek, May 2020
